# Ultrafast photophysics of a positive reversibly switchable fluorescent protein

**DOI:** 10.1039/d5sc04491j

**Published:** 2025-08-07

**Authors:** Anam Fatima, YongLe He, James N. Iuliano, Gregory M. Greetham, Partha Malakar, Christopher Hall, Helena A. Woroniecka, Brian C. Richardson, Jarrod B. French, Andras Lukacs, Peter J. Tonge, Stephen R. Meech

**Affiliations:** a School of Chemistry, University of East Anglia Norwich NR4 7TJ UK s.meech@uea.ac.uk; b Department of Chemistry, Stony Brook University Stony Brook New York 11794 USA peter.tonge@stonybrook.edu; c Central Laser Facility, Research Complex at Harwell, Rutherford Appleton Laboratory Didcot OX11 0QX UK; d Department of Biophysics, Medical School, University of Pecs 7624 Pecs Hungary Andras.lukacs@aok.pte.hu; e The Hormel Institute, University of Minnesota Austin MN 55912 USA; f School of Chemistry, University of Melbourne Parkville VIC 3010 Australia; g Department of Biomedical Genetics, University of Rochester Rochester NY 14642 USA

## Abstract

Reversibly switchable fluorescent proteins (rsFPs) are essential tools in super-resolution imaging. The mechanism operating in the widely applied negative switching rsFPs has been studied in detail. Much less attention has been paid to the positive switching rsFP variants, which offer the potential benefit of emissive states that do not photoswitch during measurement. Here we probe photochemical mechanism in all three photoactive states of the positive switching rsFP Kohinoor using a combination of ultrafast transient absorption, to probe chromophore population dynamics, and time resolved infrared, to access both chromophore populations and their effect on the surrounding protein matrix. We establish that none of the photochemical reactions are simple rate processes with transient absorption and transient IR data characterised by a common two component relaxation mechanism. Transient IR measurements reveal instantaneous coupling between the electronically excited chromophore and its protein environment, indicating that coupling arises from electrostatic or H-bonded interactions. In both on- and off-switching states the early phase of the excited state dynamics involve an initial relaxation in the perturbed protein environment, which leads to an intermediate state from which chromophore isomerization occurs. This result suggests that the protein dynamics play an active role in steering the excited state reaction, which is in-turn consistent with the known key role of the protein environment in tuning FP photophysics. Identifying and modifying the interactions between chromophore and protein will provide a means to optimise rsFP performance and thus a basis for development of improved labels for super-resolution bioimaging.

## Abstract 2

Kohinoor is a positive reversibly switching fluorescent protein. Ultrafast electronic and vibrational spectroscopy suggest that the chromophore switching mechanism is steered by dynamics in surrounding protein residues.

## Graphical abstract

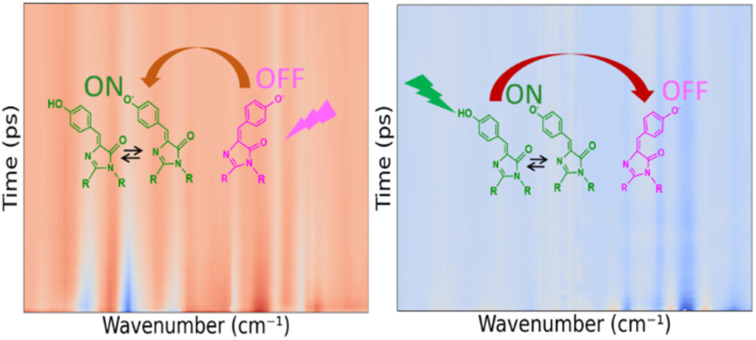

## Introduction

The discovery and development of fluorescent proteins (FPs) drove multiple advances in live cell bioimaging. The original isolation and characterization of the green FP (GFP) from *Aequorea victoria* (avGFP) was followed by rounds of mutagenesis to develop FPs suitable for protein-specific multicolour imaging and co-localisation experiments.^1–3^ A significant advance was the serendipitous discovery of photoconvertible FPs such as kaede, isolated from stony corals, which permit “optical highlighter” imaging of photoselected populations.^4–8^ This was followed by discovery of the reversibly switchable (rs) FP Dronpa.^2,9–13^ The ability to repeatedly switch Dronpa between a highly fluorescent on-state and a dark off-state by successive visible and UV irradiation was central to the development and exploitation of super-resolution bioimaging. Several other rsFPs were developed with different brightness, maturation and switching rates, as required to match particular imaging methods.^14–16^ At each stage in the development of FPs ultrafast time resolved spectroscopy was critical in unravelling the underlying photophysics, which, when coupled with quantum and molecular dynamics calculations, provided insight into mechanism and suggested routes to the rational design of improved rsFPs.^17–28^

**Fig. 1:**
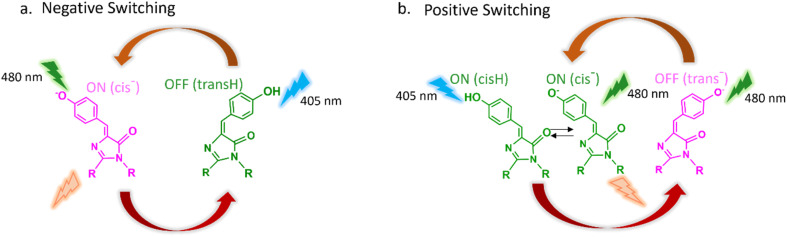
Isomeric form and protonation state for rsFP between on (pink) and off (green) states. (a) The two-state negative switching rsFPs exemplified by Dronpa contrasted with (b) the three state positive photoswitching rsFPs of the Padron type.

The mechanism of photochromism in Dronpa, its mutants and other rsFPs was characterised by determination of off- and on-state structures,^12,29,30^ time resolved optical and vibrational spectroscopy,^20,31–33^ and femtosecond serial crystallography.^17,18,24,34^ The off to on reaction is initiated by UV excitation of the *trans* protonated isomer of the chromophore (*trans*H, Fig. 1a). This results in an excited state structure change followed by internal conversion to the ground state where further structural reorganisation in both the chromophore and its environment yields a protonated *cis* isomer (*cis*H) on the ps – 100 ns timescale with a *ca* 10% quantum yield. This is followed on the tens of μs timescale by ground state deprotonation to form the on-state, an anionic *cis* chromophore (*cis*^−^).^20,33^ Visible irradiation of this on-state yields the fluorescence required for imaging but also leads to reverse photochemical isomerization and reprotonation to reform the *trans*H off-state (Fig. 1a). One consequence of this is that the emission of Dronpa necessarily fades during measurements at a rate which is not under independent experimental control, limiting the use of rsFPs in some applications. Members of the Dronpa family are referred to as the negative switching rsFPs (Fig. 1a).

Jakobs and co-workers demonstrated that only two mutations around the chromophore in Dronpa led to Padron, the prototype of a group of rsFPs in which the resting nonemissive off-state is the *trans* anionic form of the chromophore (*trans*^−^) which absorbs in the visible.^35^ Irradiation of *trans*^−^ at 480 nm was shown to convert the off-state to an on-state comprising an equilibrium between *cis*H and *cis*^−^ (Fig. 1b). Continued irradiation at 480 nm yields steady emission from *cis*^−^, which does not photoconvert back to the off-state. Only irradiation into the UV absorbing *cis*H (*e.g.* at 405 nm) results in photoisomerization and deprotonation to reform the *trans*^−^ off-state (Fig. 1b). The much smaller family of rsFPs exemplified by Padron are referred to as the positive switching rsFPs. The separation of emission from photoswitching enabled in positive switching rsFPs offers benefits for imaging modalities,^2^ such as RESOLFT^36,37^ and polarization modulation super-resolution microscopy,^38^ while the separation of on- and off-switching is likely to be useful in any two-colour imaging methods.^35,39^ However, applications of positive rsFPs have been limited in part due to low photostability and brightness.

In contrast to the negative switching variants the mechanism operating in positive switching rsFPs has been little investigated, with only two structural and two ultrafast transient absorption (TA) studies of Padron reported.^40–43^ In this work we apply ultrafast time resolved infrared (TRIR) along with TA and time resolved fluorescence (TRF) to characterise in detail the photodynamics in all three chromophore states (*cis*^−^, *cis*H, *trans*^−^) of the positive switching rsFP Kohinoor. Kohinoor was developed by mutagenesis of Padron to give a brighter, faster switching rsFP;^37,38^ further improvement in this direction has been reported with the development of Kohinoor2 (ref. 38) and Padron2.^36^ Our aim is the resolution of a detailed switching mechanism in positive rsFPs to support rational design of proteins with an optimised switching yield and brightness.

## Results and discussion

The paper is constructed as follows. The first section sets out Kohinoor structure and steady state electronic spectra which provide a level scheme to label the switching states and transitions under investigation. Each state is then studied in-turn by TA and TRIR. These two measurements allow us to correlate chromophore evolution (TA) with perturbations to protein structure (observed in TRIR). We show that in each case chromophore electronic excitation perturbs the protein environment, and that the subsequent TA and TRIR data share a common two-step relaxation mechanism and timescale. The first step in the mechanism of the on- and off-switching transitions is dominated by relaxation of the perturbed protein response. This result suggests a key active role for the protein environment in steering the rsFP switching reactions, as is discussed in detail in a final section.

### Steady state structure and spectroscopy

**Fig. 2:**
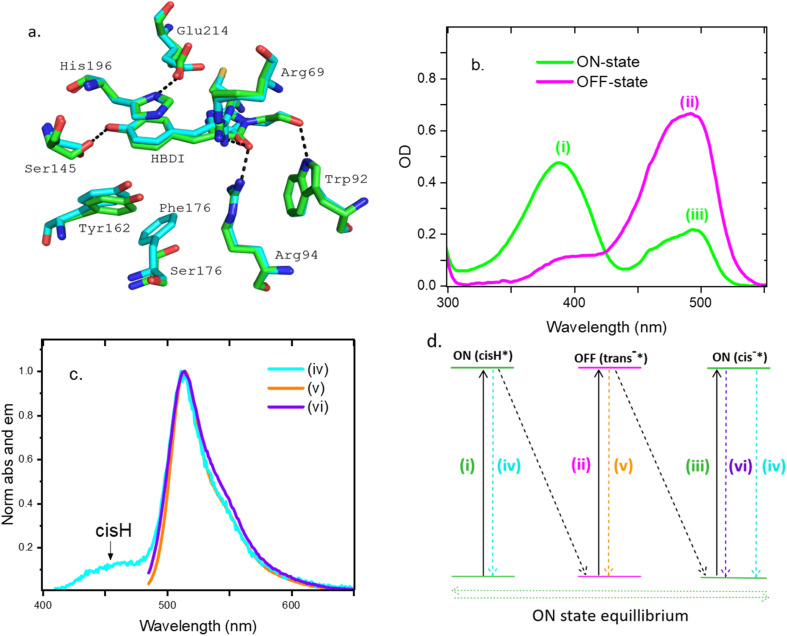
(a) Structure of Kohinoor (cyan) in its on-state (pdb 9DVE) compared with Padron0.9, pdb 3ZUJ (green). The amino acid numbers correspond to Padron. (b) Steady state absorption spectra of the on (*cis*H/*cis*^−^ equilibrium) and off (*trans*^−^)-states normalised to protein absorbance at 280 nm (c). Peak normalized steady state emission spectra of the on excited at 480 (*cis*^−^, purple) and 400 nm (mainly *cis*H, cyan) and the off (480 nm, mainly *trans*^−^, brown) states of Kohinoor in water (d) a level scheme showing the electronic states of the chromophore accessed in Kohinoor and marking the absorption (2b) and emission (2c) transitions between them. Dynamics following each excitation transition are discussed in turn in the following sections, which will establish that each chromopore level structure shown must be adapted to include their specific interactions with the protein.

The crystal structures of Padron0.9 (a variant of the original Padron) in both on- and off-states were measured by Brakemann *et al.*, revealing *cis*(*trans*) isomers of the chromophore are present in the on(off) states respectively.^41^ Comparison of the two structures showed minimal reorganisation of the amino acid residues surrounding the chromophore between these isomers (in contrast to the negative switching Dronpa, where reorganization in the protein environment is extensive^12,29^). The structure of Kohinoor had not been reported. For completeness we solved its structure in the on-state, (PDB 9DVE see electronic supplementary information, ESI, and Table S1 for additional data) and compared it with Padron0.9 (Fig. 2a). The chromophore structures are similar but not identical and there are also subtle differences in the wider structure. These small differences deserve comment in the light of our observation of differences in the photophysics between Kohinoor and Padron in the on-state (see below). Comparing the two structures (Fig. 2a), the F176S mutation in Kohinoor reshapes the region around the chromophore (see also Fig. S14). The loss of the bulky aromatic group apparently increases solvent accessibility and relaxes the steric bulk that contributes to the positioning of the neighboring Y162. The resulting shift in Y162 allows S145 to adopt a more favorable interaction with the chromophore, with a reduced hydrogen bond distance of 2.7 Å in Kohinoor, compared to 3.0 Å in Padron0.9, and a more favorable bond angle. The more open local environment also allows for additional conformational flexibility of the chromophore (Fig. S14) which can therefore adopt a slightly different conformation compared to Padron0.9 (Fig. 2a and S14) most notably in the methylene bridge region. Changes in that region are known to modify excited state dynamics in other FPs and in the bare chromophore.^44,45^

Absorption spectra of Kohinoor's on and off-states are shown in Fig. 2b. The *trans*^−^ off-state spectrum is dominated by a band at 490 nm; the high energy shoulder (*ca* 400 nm) is of variable amplitude and therefore probably due to unconverted *cis*H on-state. The on-state formed by irradiation of *trans*^−^ has two bands at 388 nm and 494 nm assigned to *cis*H and *cis*^−^ respectively. That these two bands are in equilibrium rather than arising from two uncoupled protein structures is demonstrated by their pH dependence (Fig. S13). A similar equilibrium exists in avGFP. The emission spectra arising from excitation of the two on-state bands were measured (Fig. 2c). Excitation of *cis*^−^ at 480 nm gave strong emission with a maximum at 510 nm and showed poorly resolved vibronic structure, which is characteristic of *cis*^−^ FP emission. Excitation of *cis*H at 400 nm revealed emission weaker by a factor of >100 but still dominated by the characteristic *cis*^−^ emission, peaking again at 514 nm, but now with a blue shifted shoulder in the range 420–480 nm. The latter blue emitting state is characteristic of the weak broad unstructured emission of *cis*H, previously resolved in avGFP and its mutants.^46,47^ The dual emission spectrum of *cis*H observed here can in principle arise from either excited state proton transfer (ESPT), as in avGFP,^27^ or from excitation of a mixture of *cis*H/*cis*^−^ ground state species (as in S65T GFP at pH 5.5 (ref. 46)). Excitation spectra (Fig. S1) reveal that the 460 nm emission comes exclusively from excitation of *cis*H while the emission at 530 nm is exclusively from excitation of *cis*^−^; there is no discernible contribution from ESPT. Evidently while 400 nm excitation is dominated by the *cis*H absorption a small population of the strongly emissive *cis*^−^ is also excited. This suggests that there is no wavelength where *cis*H can be exclusively excited in Kohinoor. We return to this point in the discussion of time resolved data below. Finally, the fluorescence spectrum measured after 480 nm excitation of the *trans*^−^ off-state has a profile similar to *cis*^−^ with a maximum at 510 nm but with less well resolved vibronic structure, is much weaker (by a factor >20, Fig. S2) and is slightly broader; some unconverted *cis*^−^ may also contribute in this region.

### Off- to on-state reaction

**Fig. 3:**
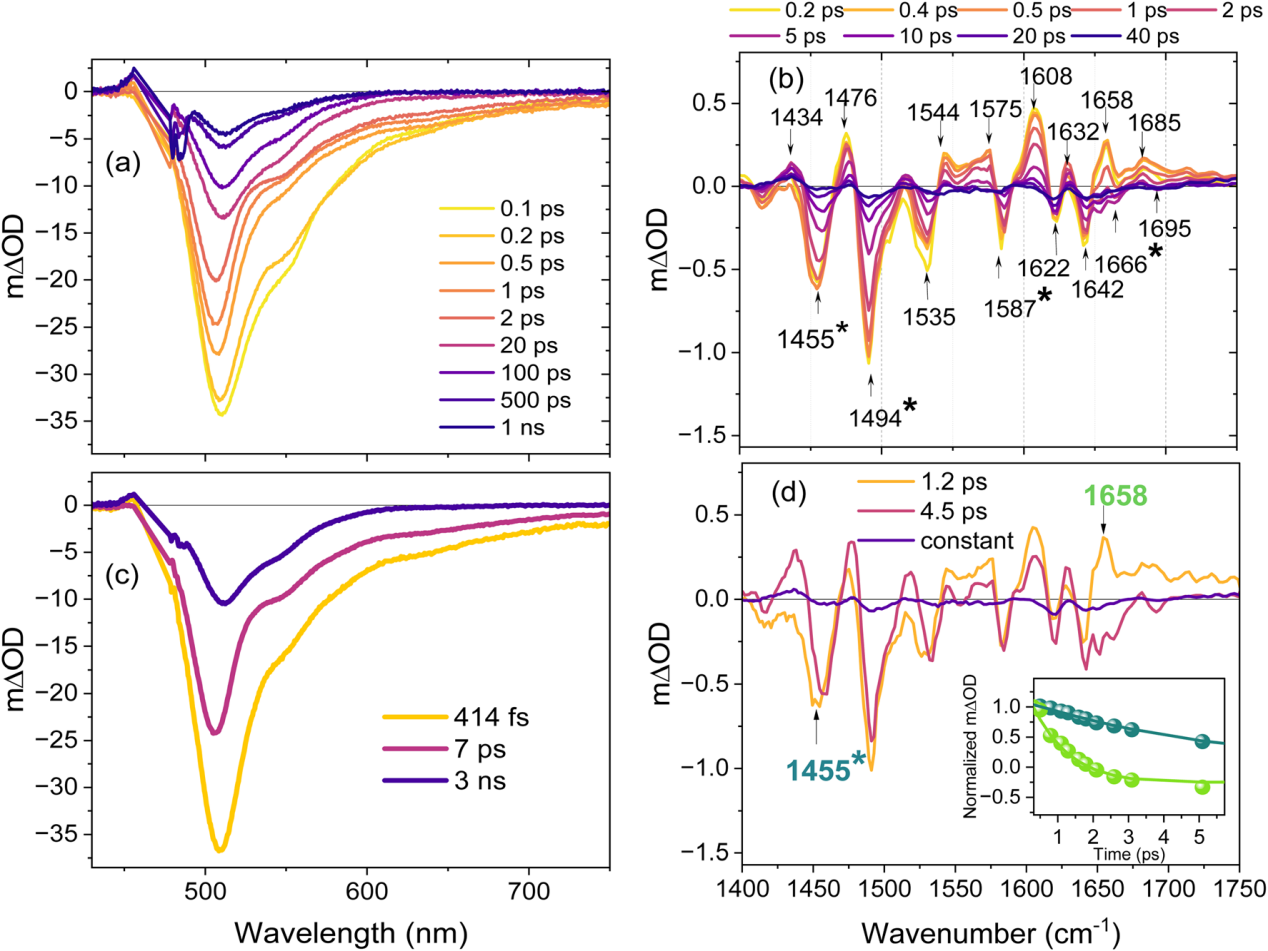
(a) Transient absorption spectra of Kohinoor obtained by 465 nm pump pulse excitation of the off-state (*trans*^−^) in H_2_O; oscillations below 500 nm arise from pump pulse scatter. (b) TRIR spectra recorded after excitation of the off-state at 475 nm in D_2_O. The asterisk marks bleach modes that are mainly associated with the chromophore – see text. (c) EADS obtained from the global analysis of the TA data using the sum of two decay components (assuming a sequential kinetic model) and a fixed 3 ns final component and (d) EADS obtained by global analysis of the TRIR data using the same model as (c) with a constant final component; note that the final very low amplitude component continues to evolve on a tens of ps timescale (see below). The inset shows intensity normalized temporal evolution of modes identified as protein and chromophore (the latter labelled *) to highlight their different relaxation times; the solid lines are from the global fit. The samples were measured in a flow cell and the reservoir was continuously irradiated with a 405 nm LED to maintain the off-state.

Kohinoor (<1 mM at pH 8 in D_2_O buffer) was converted from the on-state equilibrium to the *trans*^−^ off-state for study by irradiation at 405 nm. The sample was flowed, and 405 nm irradiation of the reservoir was continued to maintain the off-state population during the measurements. The sample was excited with 1 kHz 100 fs pulses at either 465 nm and studied by TA (probe 450–750 nm) or at 475 nm and studied by TRIR (probe 1400–1750 cm^−1^). The two measurements allow us to correlate time dependent transient electronic and vibrational spectra; experimental data are shown in Fig. 3a and b for TA and TRIR respectively. Further experimental details are given in the SI.

The TA difference spectra (Fig. 3a) are dominated by a negative difference absorbance band which the comparison with steady state data (Fig. 2b and c) shows is a combination of ground state bleach (GSB) and stimulated emission (SE). The signal recovery is non-single exponential and incomplete in 1 ns. The initial spectral change is a fast (sub-picosecond) relaxation on the red side of the main band, assigned to evolution in SE, which increasingly dominates the signal for wavelengths greater than 500 nm, Fig. 2b and c. This relaxation leads to a narrower slightly blue shifted negative absorbance difference band which then relaxes in a few ps to yield a long lived (*i.e.* >1 ns lifetime) spectrum which is slightly red shifted and structured. This low amplitude final long-lived SE component is assigned to an emissive population which may be either residual on-state (as noted in steady state fluorescence, Fig. 2b) or a long lived population of off-state; these possibilities cannot be resolved from the present data.

Global analysis of the TA data assuming a sequential kinetic model leads to the evolution associated difference spectra (EADS) shown in Fig. 3c (the corresponding decay associated difference spectra, DADS, from a parallel decay model are shown in Fig. S3 together with data showing quality of fit). In principle the sequential and parallel schemes are not readily distinguished by the TA data alone. The rationale for preferring the sequential model are set out below where TRIR are considered. A two-state (sequential) relaxation mechanism to reach a final state is the simplest model which accurately describes the data (it does not preclude more complex models). The analysis shows that the initial spectral evolution occurs in 0.41 ps followed by a 7 ps overall decay to leave the long-lived contribution, set here to 3 ns (approx. lifetime of *cis*^−^) but values from 1 to 10 ns made no difference to the EADS. This is consistent with an initial rapid relaxation in the SE profile of *trans*^−^ followed by a 7 ps population decay of the relaxed state. The 7 ps population decay mainly repopulates the initial *trans*^−^ ground state. This is consistent with the low (*ca* 1%) off to on quantum yield of Kohinoor.^36^ These data are qualitatively similar to those of Walter *et al.* for Padron0.9 at pH 10, although the measured TA profiles are different.^43^

Mechanistic details of the off-state excited state dynamics are revealed by TRIR (Fig. 3b). The IR spectra of the *cis*^−^ and *trans*^−^ forms of the chromophore were previously calculated by DFT and measured in buffer (the calculated spectra are shown in Fig. S4). The isolated chromophore has four prominent ground state modes in the TRIR range studied; the assignments and spectral shifts between *cis*^−^ and *trans*^−^ are given in Table S2. The initial 0.2 ps TRIR difference spectrum is markedly more complex than predicted by this calculation, with at least seven bleach bands (negative ΔOD) observed, some with complex asymmetric profiles suggesting multiple underlying contributions. In addition, there are several transients (positive ΔOD signals) none of which are seen in the TRIR of the isolated chromophore.^48^ This result indicates significant coupling between *trans*^−^ and surrounding amino acid residues, such that the chromophore localised excitation, which initiates the isomerization reaction, instantaneously perturbs the vibrational spectra of adjacent amino acids.

The evolution of the TRIR spectra is wavenumber dependent. In the 1640–1700 cm^−1^ region, where the only chromophore ground state mode predicted is the C=O stretch (Fig. S4), there is an instantaneous bleach with a complex profile peaking at 1642 cm^−1^ and two promptly formed transients at 1658 and 1685 cm^−1^. Based on the calculations, the measured TRIR of other FPs and of the isolated chromophore (Table S2), the bleach wavenumber is too low for the *cis*^−^ chromophore C=O, which is likely weak and hidden under the transient, so the 1642 cm^−1^ signal is assigned to a perturbed protein residue. The transient at 1658 cm^−1^ relaxes in <2 ps to leave a weak but reproducible transient/bleach pair at 1685/1695 cm^−1^ and reveals a bleach at 1666 cm^−1^, which is more consistent with the chromophore ground state C=O mode (Table S2). The intermediate state formed in <2 ps subsequently decays on a several ps time scale. The initial fast relaxation phase is evident elsewhere in the TRIR, especially in the broad complex transient between 1540 and 1580 cm^−1^, the lineshape evolution of the 1455 cm^−1^ bleach and the appearance of a transient at 1434 cm^−1^. The intense 1494 and 1455 cm^−1^ bleaches appearing at *t* = 0 can be assigned to chromophore phenyl modes. The latter is calculated to have greater relative intensity in *trans*^−^ compared to *cis*^−^ (Fig. S4) consistent with these observations. Finally, it is interesting to note that the extensive perturbation of protein modes induced by chromophore excitation revealed by TRIR is in contrast to the minimal structural perturbation of surrounding residues due to isomerization shown in the crystal structures of Padron.^41^ Evidently the excited state dynamics are influenced by interactions which are either transient or not resolved in the nuclear structure.

The TRIR data are well fit by the same sequential two step plus long-lived state model used for TA, and the resulting EADS are shown in Fig. 3d (DADS and fit quality are presented in Fig. S5). The time constant for the fast initial formation of the intermediate is 1.2 ps, which then relaxes to repopulate the ground state (*e.g.* filling the chromophore bleach signals at 1494, 1587 and 1666 cm^−1^) in 4.5 ps. From the experimental TRIR data and EADS it is evident that the fast initial phase is a relaxation which does not repopulate the ground state. This argues against the parallel decay model in which there are two (or a distribution) of distinct ground states with distinct spectra decaying to the ground state with different rates. This is not observed, and the data suggest an initial relaxation in the excited state occurring before relaxation/isomerization. We thus assign the transient at 1658 cm^−1^ to relaxation of the *trans*^−^ excited state coupled to its protein environment. This relaxation forms the intermediate chromophore structure characterised by bleach and transient features between 1670 and 1720 cm^−1^; the transient is at too high a wavenumber for a *trans*^−^ chromophore mode (Fig. S4) so is assigned to a structurally distorted excited state. The 1695 cm^−1^ bleach must arise from a perturbed protein mode; the most likely candidate in the region of the chromophore is Glu214.

The formation of excited state intermediates is a feature of photoisomerization reactions. Such intermediates often have a twisted structure with respect to the initial state and reduced transition moment to the ground state.^49,50^ We thus correlate the 1.2 ps structural evolution with the 0.4 ps relaxation in SE observed in the TA (Fig. 3a) and assign it to excited state structure evolution of the chromophore coupled to its protein environment. Crucially the principal changes observed in TRIR during this initial 1.2 ps phase involve evolution in the protein modes perturbed on excitation (*e.g.* the transient above 1640 cm^−1^, the broad transient around 1540–1580 cm^−1^, and the complex profile of the chromophore bleach at 1455 cm^−1^ which reflects the formation of the transient at 1434 cm^−1^). In contrast, the chromophore bleach modes (1455 and 1494 cm^−1^) are unchanged during this initial phase (Fig. 3b and d and inset). Likely contributions to the observed evolution in protein modes include the strong interaction between *trans*^−^ and the tyrosine residue (Tyr162) which plays a key role in stabilizing the off state (Fig. 2a).^41^ The Tyr residue has phenyl stretching modes near 1500 and 1600 cm^−1^, which may contribute to the complex TRIR profile in that region. However the adjacent Glu and Arg residues (Glu214 and Arg69) may also play a role;^51,52^ more definitive assignment requires TRIR of isotope edited proteins.^20^

The 1.2 ps relaxation time recovered from TRIR is longer than for TA (0.4 ps), and the difference is outside experimental error. Such differences can arise as a result of a decrease in the S_1_ → S_0_ transition moment along the reaction coordinate leading to the intermediate. This time dependent decrease will lead to the SE (observed in TA) relaxing faster than the excited state population (measured in TRIR); a similar result has been observed in studies of other excited state isomerization reactions.^53^ The central result here is that the protein dynamics observed in TRIR correlate with the initial step in the evolution of the chromophore SE, suggesting an active involvement in the excited state relaxation.

The intermediate formed in 1.2 ps relaxes to the original *trans*^−^ ground state in 4.5 ps, which we associate with the 7 ps component observed in TA. Since SE dominates the TA signal on this longer time scale (Fig. 3a) the intermediate must be formed in an electronically excited state. From this we infer that it cannot be a 90° twisted form of the chromophore (which is the calculated minimum energy structure in the excited state of the bare chromophore) since this would have both a sub-ps lifetime, due to a minimum energy conical intersection (CI) with the ground state at that geometry, and a negligible transition dipole moment for SE.^44,54,55^ Instead, the present data suggest that the intermediate state is formed from an initial disruption of protein chromophore interactions occurring in 1.2 ps in the excited electronic state to form a distorted chromophore structure with reduced S_1_ → S_0_ transition moment, from which the chromophore accesses the CI with the ground state in a few ps.

The ‘long-lived’ spectrum formed in 4.5 ps recovered from global analysis of the TRIR data (Fig. 3d) in fact relaxes to the baseline on a longer time scale of tens of ps (resolved in the experimental data Fig. S6) but the change in ΔOD is so small that the global analysis does not capture it, even when an additional component is added. We suggest that this tens of ps final recovery phase is a relaxation in the ground state formed initially with a chromophore structure that is out of equilibrium with its protein environment, *i.e.* the original ground state protein structure changed following excitation during the 1.2 ps phase, and the reverse relaxation once the chromophore ground state is restored takes tens of picoseconds. The true final TRIR spectrum is mainly baseline with small, broad but reproducible signals around 1460 cm^−1^ and at 1650 cm^−1^ (Fig. S6). These very small residual perturbations must arise from the *ca* 1% yield of the *cis*^−^ photoproduct, which will ultimately establish the equilibrium population of *cis*H.

### On- to off-state reaction

**Fig. 4:**
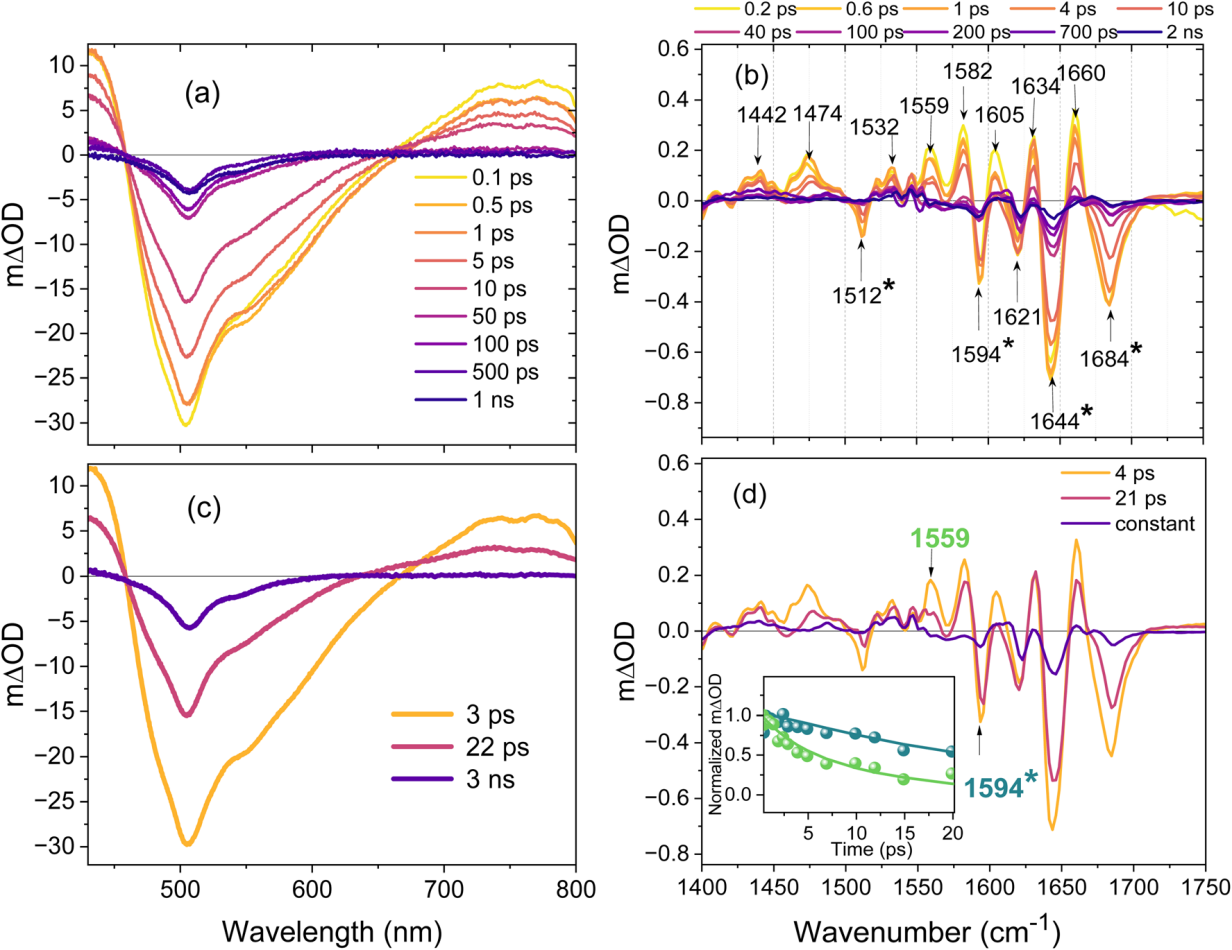
(a) Transient absorption spectra of Kohinoor obtained by 400 nm pump pulse excitation of the on-state (*cis*H) in H_2_O (b) TRIR spectra recorded after excitation of the on-state at 380 nm in D_2_O. The asterisk marks bleach modes that are mainly associated with the chromophore – see text. The fit lines are from the global analysis to the entire data set. (c) EADS obtained from the global analysis of the TA data using the sum of two decay components (assuming sequential kinetic model) and a 3 ns decay (d) EADS obtained by global analysis of TRIR data. The inset shows intensity normalized temporal evolution of modes identified as protein and chromophore (*) to highlight their different relaxation times. The solid lines are from the global fit. The samples were measured in a flow cell and the reservoir was continuously irradiated with a 505 nm LED to maintain the on-state.

To probe the on- to off-state reaction the pump excitation wavelengths were either 400 nm (TA and TRF) or 380 nm (TRIR). Both wavelengths directly excite the *cis*H population which is in equilibrium with the *cis*^−^ state, which together constitute the on-state. The existence of a *cis*H/*cis*^−^ equilibrium rather than two isolated states is demonstrated by the observed pH dependence (Fig. S13) as seen previously in Padron.^41^ Excitation of *cis*H leads to the *trans*^−^ off-form of the chromophore, with a higher (*ca* 8%) yield than for the off- to on-state transition.^41^ Again, the sample was flowed, and the reservoir continuously illuminated at 505 nm to maintain the on-state population; the experimental data are shown in Fig. 4a and b for TA and TRIR, respectively.

Although broader and blue-shifted, there is a superficial similarity of the TA data in Fig. 4a to that in Fig. 3a. This is misleading, the narrow profile resolved for *cis*H SE arises from an overlap of its broad structureless SE with the pair of excited state absorption (ESA) bands seen at long and short wavelengths, leading to the narrow asymmetric SE profile observed (the *cis*H GSB expected on the blue side is obscured by the ESA). Similar spectra have been reported elsewhere for *cis*H.^33,56^

The amplitude of the *cis*H SE/TA in Kohinoor decays monotonically. Surprisingly, this is in marked contrast to the TA for the structurally and spectroscopically very similar Padron0.9 studied previously by Walter *et al.*^43^ They observed a ps rise in SE, assigned to formation of *cis*^−^ in the excited electronic state by an ESPT reaction. Such an ultrafast ESPT is well characterised in avGFP^27,28^ and was also observed in Padron in the study of Fron *et al.*^42^ Further, the risetime in the SE was shown to become longer when the experiment was performed in D_2_O, another characteristic of a proton transfer reaction. In contrast, neither the TA nor the steady state data for Kohinoor (Fig. S1) show any evidence for ESPT. In particular, the SE in Fig. 4 rises promptly on excitation and is characteristic of the *cis*H state (see above).

Walter *et al.* studied Padron0.9 at pH 10.^43^ To investigate this anomaly further steady state measurements for Kohinoor were repeated at pH 10 but no evidence for ESPT was found (indeed *cis*H was barely resolved, Fig. S7). Further, we measured TRF of Kohinoor (pH 8) on 400 nm excitation with sub 100 fs time resolution (Fig. S8). TRF was observed at emission wavelengths of 460 nm and 510 nm (*i.e.* in the two emission bands observed, Fig. 2c). The former shows bi-exponential decay with sub-ps and 9 ps decay components. The longer wavelength was best fit by three exponential decaying components with decay times of 1 ps and 20 ps and a >500 ps component of low amplitude (Table S3), the latter probably arising from excitation of the underlying *cis*^−^ population (see also Fig. 2c). Significantly, no ps risetime was resolved at 510 nm, ruling out ESPT in Kohinoor. Thus, despite structural (Fig. 2a) and spectroscopic similarities Kohinoor and Padron0.9 show distinct differences in photochemical mechanism.

We suggest that these differences arise because of the small changes is structure reported in Fig. 2a. For example, the F176S mutation and the shifts in Y162 and S145 will change the protein environment around the phenolic OH, potentially suppressing ESPT in Kohinoor. Previous steady state data also suggests significant differences between the two proteins (some of these differences are tabulated in Table S4). The p*K*_a_ for Padron0.9 and Kohinoor are different, probably as a result of its sensitivity to the charges on amino acids, which themselves vary with pH.^38,43^ Further, the structure (Fig. 2a) revealed small changes in chromophore geometry, which result in modest shifts in electronic spectra between Padron and Kohinoor,^36^ and could modify excited state photoacid properties, 
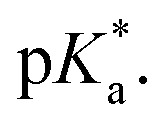
 The ESPT reaction in Padron0.9 and its variants will be the topic of a separate study by TRIR. Here we simply note that ps ESPT must occur in competition with the isomerization required for off-state formation, which may explain the much lower quantum yield for on- to off-state photoswitching in Padron0.9 compared to Kohinoor.^36^

The TA data (Fig. 4a) were subjected to global analysis and a good fit was again achieved with the two sequential steps plus a long-lived component model; additional components slightly improved fit quality but provided no new physical insight. The EADS are shown in Fig. 4c (DADS and fit quality are shown in Fig. S9) and report 3 ps and 22 ps time constants. The amplitude decays monotonically but the EADS narrow between initial and intermediate states, with SE amplitude on the red side decreasing along with a decrease in ESA. The intermediate formed in 3 ps decays to the long-lived final EADS, which is narrow and slightly structured with a profile similar to the SE of the *cis*^−^ state; this is consistent with excitation of the previously identified minor *cis*^−^ population absorbing at 400 nm (Fig. 2b and c). Because of its long lifetime this component will make a constant contribution to the transient measurements.

The TRIR data (Fig. 4b) are well resolved but complex. The four bleaches at 1684, 1644, 1594 and 1512 cm^−1^ can be assigned as chromophore ground state modes; similar bands were observed in a study of *cis*H in avGFP.^57^ The band at 1621 cm^−1^ and most of the transient features are specific to the chromophore in its protein environment so are assigned to chromophore protein interactions perturbed on electronic excitation. The dominant feature in the TRIR evolution is the decay of excited state transients and simultaneous recovery of the ground state bleaches. However, recovery is incomplete within 2 ns, pointing to a long-lived change in the vibrational spectrum. In addition, there is an initial relaxation phase which is most prominent in the blue shift in the 1474 cm^−1^ transient and fast decay at 1559 and 1605 cm^−1^ (Fig. 4b). These bands have no counterpart in the spectrum of the isolated chromophore,^48^ nor is anything similar observed in the TRIR of *cis*H in avGFP or *trans*H in Dronpa,^20,57^ so they are assigned to specific *cis*H protein interactions in Kohinoor.

The two step EADS model (DADS and fit quality are in Fig. S10) yields time constants of 4 ps and 21 ps, which align well with the 3 ps SE narrowing and 18 ps decay observed in TA, suggesting they reflect the same underlying processes. The EADS show that the signals assigned to perturbation of the IR spectrum of protein residues relax mainly in the first EADS, while chromophore ground state recovery is dominant in the second step (Fig. 4b and d and insert). This suggests a mechanism in which electronic excitation gives rise to an initial change in the chromophore's interaction with its matrix that facilitates motion along the isomerization coordinate *i.e.* the interaction with the protein which relax in the first phase plays an active role in the isomerization reaction. The overall reaction then mainly leads to repopulation of the *cis*H ground state with a minor route to the *trans*^−^ product, presumably *via* a CI encountered near a twisted geometry.

Both experimental TRIR and global analysis reveal a long-lived low amplitude component, which was probed on the ns–μs timescale (Fig. S11). Unlike the off- to on-state, this component does not recover in ns but persists for at least 1 μs. There is a bleach/transient pair at 1689/1672 cm^−1^ (obscured in Fig. 4 but resolved in Fig. S11) which we assign to *trans*H formation, where the transient has the a small downshift in wavenumber in *cis*H to *trans*H isomerization as predicted by calculation.^20^ This signal does not evolve further in at least 1 μs. We note that the rather clean excited state isomerization suggested by these data and the crystal structures^41,58^ suggests Kohinoor as a good candidate for study by serial femtosecond crystallography. There is weak evidence for further very slow evolution in the TRIR associated with the *trans*H product, with amplitude in a transient at 1607 cm^−1^ and a bleach at 1593 cm^−1^ both decreasing slightly on the longer time scale. This observation may indicate further reorganisation in the protein environment. Significantly, we do not observe formation of a strong transient near 1500 cm^−1^ which would indicate formation of *trans*^−^, suggesting that the deprotonation required for off-state formation occurs on a longer (>1 ms) timescale.

Finally in this section it is interesting to compare the TRIR observed for *cis*H excitation here with the same form of the chromophore excited in avGFP and in Dronpa. The early time TRIR are compared as the later time spectra will reflect the different photochemical fates (ESPT and isomerization respectively). avGFP and Dronpa were compared previously.^21^ In both cases the TRIR are more complex than for the chromophore alone, indicating the presence of coupled chromophore–protein modes in all cases. The spectra of avGFP and Dronpa are similar, but with marked differences between amplitude in some modes, ascribed to different H-bonded interactions. Further, Dronpa showed larger amplitudes in coupled chromophore–protein modes than avGFP, with more marked ‘transient’ (positive ΔOD) contributions. Comparing data for *cis*H in Dronpa and Kohinoor, the TRIR share the same features with major contributions from protein coupled modes. However, more structure is resolved in Dronpa,^20^ which is consistent with the more complex protein environment, most notably the Arg residue (Arg69) which has to move significantly between Dronpa off and on-states, but which does not play a role in Kohinoor. Further, the spectral changes over time show different behaviour, consistent with the different outcomes for negative and positive switching rsFPs, specifically in Kohinoor we find a distinct separation in time scale for the relaxation of the coupled protein–chromophore modes and the excited state population decay.

### Fluorescent on-state dynamics

**Fig. 5:**
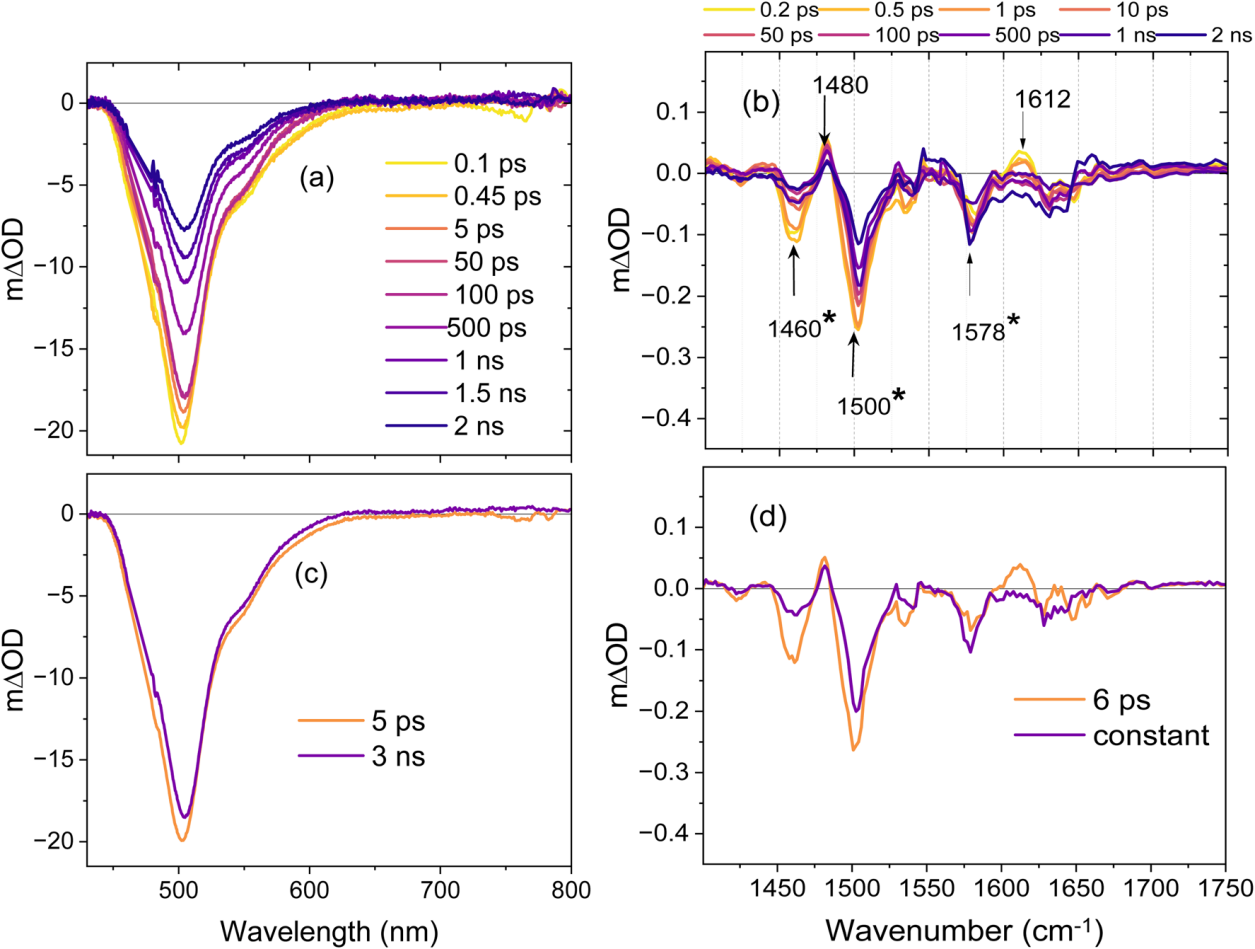
(a) Transient absorption spectra of Kohinoor obtained by 475 nm excitation of the on-state (*cis*^−^) in H_2_O. Data are presented out to 2 ns to reflect the slower relaxation of the dominant emissive *cis*^−^ state. (b) TRIR spectra recorded after excitation of the on-state at 475 nm in D_2_O. The asterisk marks bleach modes that are mainly associated with the chromophore – see text. (c) EADS obtained from the global analysis of the TA data using one decay component and a constant 3 ns decay (d) EADS obtained by global analysis of TRIR data.

The on-state was prepared as described above but the pump wavelength was adjusted to 475 nm to pump the *cis*^−^ population of the on-state equilibrium. The expectation was that this state would simply relax mainly by emission with the previously measured 3.5 ns lifetime. However, TRIR reveals underlying ultrafast dynamics even for this state. The TA and TRIR data are shown in Fig. 5a and b.

The TA data are peaked at 500 nm, which shows that both GSB and SE contribute, with no ESA observed. The SE shows the long lifetime and weakly resolved vibronic structure expected from the steady state fluorescence (Fig. 2c). The TA dynamics are not strictly exponential, and a sequential two-step model shows a small evolution which gives rise to a modest red-shift in 5.3 ps followed by the expected on-state ns decay time (Fig. 5c). Thus, although there are two distinct and different decay times the associated emission spectrum is, aside from the small spectral shift, the same for both and characteristic of *cis*^−^. The TRIR data are complex showing fast bleach recovery and, unusually, a bleach apparently growing in at 1578 cm^−1^. Global analysis of these TRIR data reveals 6 ps and long-lived (>1 ns) contributions, in good agreement with TA dynamics. The EADS are in Fig. 5d and the DADS and fit data are in Fig. S12. The EADS suggest an inhomogeneous decay. There is a clear fast decay in a transient at 1612 cm^−1^ with associated bleach recovery in chromophore modes at 1500 and 1460 cm^−1^. Both these chromophore modes are blue shifted from the equivalent *trans*^−^ wavenumber in agreement with calculation (Table S2). There is also a deepening of the bleach at 1578 cm^−1^ and a complex profile from 1620–1680 cm^−1^.

The two prominent chromophore bleaches (1500 and 1460 cm^−1^) recover at different rates. This result suggests two populations with fast and slow excited state decay/ground state recovery times, the slow component being the dominant form responsible for the nanosecond emission. The fast recovery component evidently has an enhanced amplitude in the 1460 cm^−1^ mode as well as a transient at 1612 cm^−1^. That this fast relaxation is associated with an increased bleach amplitude at 1578 cm^−1^ is unexpected, but we speculate that as the short-lived state recovers its ground state this results in a change in a protein residue mode which decreases in transition moment, possibly through a modification of H-bonded interactions. The difference spectrum then reports the deepening bleach observed. The complex lineshape at higher wavenumber (where only the C=O localised chromophore mode will contribute) also points to a perturbation of the protein vibrational spectrum on this timescale. The significance of the short-lived population in *cis*^−^ on-state relaxation revealed here is that it cannot contribute significantly to fluorescence, thus leading to reduced brightness. In other FPs it has been suggested that an anchoring H-bond between the phenoxy O atom and the protein matrix fixes the chromophore structure and suppresses the excited state decay of *cis*^−^,^10^ so this result may indicate co-existing populations of H-bonded and non-bonded forms of the chromophore in Kohinoor. Significantly, in the case of *cis*^−^ decay, where no switching reaction occurs, there is no evidence for a key role for the protein perturbation in the dynamics. Rather, the assignment is to an inhomogeneous ground state population of chromophore structures.

### rsFP switching and protein chromophore interactions

**Fig. 6:**
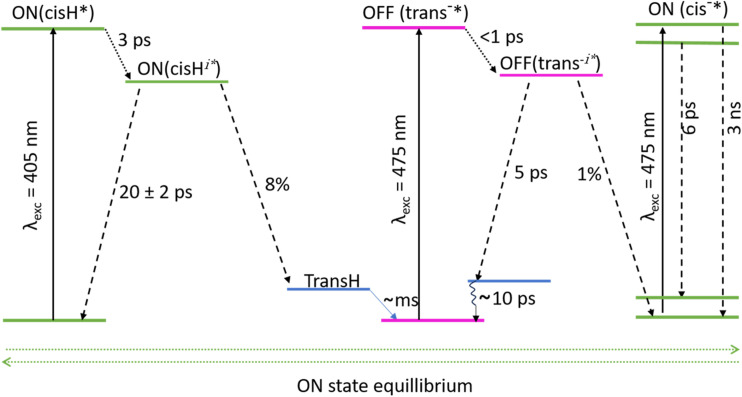
Overview of the phototransformation process in the three states of Kohinoor. Dotted arrows represent relaxation pathways of the chromophore from the Franck–Condon state (denoted by the superscript *), leading to the formation of intermediate states (denoted by the superscript *i*). Dashed arrows depict the subsequent relaxation of these intermediate excited states to the ground state(s). Excitation of the *cis*H forms an intermediate state within *ca.* 3 ps, which predominantly decays to refill the ground state in *ca* 20 ps. A minor fraction (8%) proceeds to populate the *trans*H state, which subsequently forms the *trans*^−^ on a millisecond timescale. In the off-state, following excitation of *trans*^−^ an intermediate is formed in <1 ps and primarily (99%) relaxes to a hot-ground state within in *ca* 5 ps, which then relaxes over tens of picoseconds (Fig. S6) to recover the original state (indicated by the curly arrow). The excited fluorescent on-state (*cis*^−^) undergoes an inhomogeneous decay, involving both a rapid (6 ps) excited-state decay and a long-lived fluorescent pathway (3 ns).

The excited state dynamics and mechanism for all three states of the positive rsFP Kohinoor have been investigated by application of ultrafast TRIR and TA spectroscopies. There is good agreement between the relaxation times recovered from the two experiments when a two-step sequential model is applied to the data. The resulting dynamics are summarized in Fig. 6 for all three optically accessible states, where only the very slow deprotonation of *cis*H has not been experimentally determined.

Fig. 6 shows the phototransformation mechanism connecting each of the three states of Fig. 2d. Detailed kinetic and spectral analysis showed that none of these are simple exponential rate processes. The kinetics require at least one intermediate involved in each transformation, while TRIR spectra reveal strong coupling between the chromophore and its environment, with multiple modes not associated exclusively with the chromophore contributing to the spectral evolution. In most cases these protein modes appear with the initial excitation, so they do not arise from mechanical/structural rearrangements due to changes in the chromophore's nuclear structure, such as isomerization. Rather their origin must be instantaneous changes to electrostatic or H-bonded interactions between the chromophore and its local environment. These changes must in turn reflect the different electronic structures in the ground and excited states of the chromophore. There is a growing body of evidence suggesting that such electrostatic interactions play a major role in determining FP photophysics, and the present results add to that.^59–61^

In this connection it is interesting that the structural studies showed negligible perturbation to the surrounding structure between off- and on-states,^41,58^ while the present data suggest strong interactions between the chromophore and the surrounding protein residues. This must arise from the different sensitivity of the two methods to nuclear structure and intermolecular interactions. Indeed, in terms of rsFP switching dynamics there is a similar divergence between observations by optical spectroscopy and serial femtosecond crystallography (SFX). The latter typically show a sub-picosecond appearance of either product isomers or significantly twisted chromophores formed in excited electronic states.^18,34,62,63^ In that case the chromophore dynamics are similar to theoretically predicted and experimentally observed sub-picosecond to picosecond dynamics of the isolated chromophore in the gas or fluid solvent phases.^64–67^ In contrast most optical experiments, including the current observations, show rsFP excited state relaxation on the multi picosecond timescale, forming a ground state product which evolves further in time.^20,39,62,68^ This result suggests that the protein significantly modifies the chromophore excited state reaction compared to the gas phase. As has been noted elsewhere,^34^ this divergence can likely be traced to the differential sensitivity of the two methods to nuclear structure, with optical and TRIR spectroscopy responding to electrostatic and H-bonding interactions as well as nuclear structure changes (although the assignment of a unique structure change from electronic and vibrational spectra is much less straightforward than for SFX). It would be very interesting to measure the corresponding SFX data for Kohinoor.

Here, by combining TRIR and TA measurements, a correlation between dynamics in the protein modes perturbed by electronic excitation and chromophore spectral dynamics was established. Further it was found that relaxation in protein modes occurred mainly in the initial phase of the two step relaxation. This in turn suggests that protein–chromophore interactions do not simply modify the reaction coordinate through stationary steric or electrostatic interactions, but their dynamics play an active role, relaxing to generate new intermediates in the isomerization reaction pathway (Fig. 6).

In contrast, protein modes play only a passive role in the non-switching *cis*^−^ on-state decay kinetics. It is likely that a more detailed residue specific understanding of the role of protein–chromophore interactions will require complementary ultrafast isotope labelling experiments^20^ and quantum chemical calculations on the chromophore coupled to its environment. The present results suggest that such calculations will need to incorporate more of the environment in the quantum mechanical part of the calculation than is usual. Such calculations are undoubtedly challenging but recent successful calculations on red-emitting FPs with non-reactive excited states are encouraging.^69^

Significantly, the *cis*H form of Kohinoor, which drives on- to off-state switching, was shown to behave very differently to the structurally and spectroscopically very similar Padron0.9, in that no ESPT reaction was observed in Kohinoor. This was assigned to small structural differences between Kohinoor and Padron which modify the environment of the phenolic group of the chromophore and to the different electrostatic environment and chromophore structure in the two proteins. The suppression of ESPT in Kohinoor contributes to its larger switching yield. This result further indicates how electrostatic interactions play a major role in determining FP photophysics.

While the rational design of Kohinoor variants with improved photophysical properties will be aided by the isotope labelling and QM calculations proposed above, we speculate that replacing Y162 with a smaller side chain will complement the F176S mutation and provide additional space for the *cis*–*trans* isomerization as well as increased solvent accessibility which we hypothesise will modulate the electrostatic environment of the chromophore and further improve photostability. Our analysis also suggests that replacing Y162 with a positively charged side chain that can interact with the chromophore phenol and/or S145 might further stabilize *trans*^−^ in Kohinoor and increase switching yield. Such predictions require further investigation, which is planned.

## Conclusions

The excited state dynamics in the positive switching rsFP Kohinoor have been observed. For the two switching states (*cis*H and *trans*^−^) complementary measurements of chromophore dynamics observed through TA and coupled protein chromophore dynamics measured in TRIR revealed a clear correlation between the two observations. The same two-step relaxation kinetics occur in both experiments, with TRIR revealing the first step to be dominated by evolution in the protein modes perturbed on electronic excitation. The initial excitation gave rise to instantaneous electrostatic interaction between the excited state and its environment, observed in instantaneous spectral shifts of protein modes. This initial excitation perturbation relaxes to yield a new protein–chromophore intermediate state, observed in TA measurements, which subsequently undergoes population relaxation. These observations suggest an active role for the protein–chromophore interactions in steering the photoswitching reactions, although further measurements of Kohinoor mutants together with isotope labelling are required to demonstrate this causal relationship.

Interestingly even modest changes in the protein environment (*e.g.* between Kohinoor and Padron0.9 Fig. 2a) have a profound effect on switching kinetics. These results suggest that identification of the key interactions, for example through mutagenesis and isotope editing (with Y162, E214 and R69 being key targets), will suggest routes to modifying rsFP switching pathways. The protein dynamics leading to the new protein–chromophore state are especially easy to resolve in photoactivated reactions due to the instantaneous nature of the perturbation. However, the present results suggest that any sudden change in intermolecular interaction, such as occurs in proton or electron transfer reactions, may result in fast reshaping of the environment. The resulting perturbation to the protein environment may then play a role in the subsequent reaction dynamics in ways that are not readily predicted from the crystal structure.

## Experimental

Proteins were prepared purified and characterised using standard procedures, which are detailed in the ESI. Similarly, the TRF,^70^ TA^71^ and TRIR^72,73^ methods were described previously. Important details are placed in the ESI. Global analysis used Glotaran.^74^

## Author contributions

Anam Fatima: TA and TRIR measurements and analysis. Yongle He: protein expression, sample preparation, TRIR measurement. James Iuliano: protein expression, sample preparation, TRIR measurement. Greg Greetham: construction and management of TRIR facility. Parha Malakar: construction and management of TRIR facility, support for measurements. Chris Hall: TA and TRIR measurements and analysis. Helena Woroniecka: protein expression, sample preparation. Brian Richardson: protein crystallography. Jarrod French: protein crystallography. Andras Lukacs: experiment design and conceptualization, TRIR measurement and data analysis. Peter Tonge: experiment design and conceptualization, fund raising. Steve Meech: experiment design and conceptualization, fund raising, drafting MS. All authors reviewed and edited the paper.

## Conflicts of interest

The authors declare no conflicts of interest.

## Supplementary Material

SC-016-D5SC04491J-s001

SC-016-D5SC04491J-s002

## Data Availability

Additional experimental data will be made available upon reasonable request. Supporting information presents additional details on protein preparation, X-ray structure determination (Table S1) and ultrafast measurements. Also shown are additional figures (Fig. S1–14) and tables concerning steady state electronic spectroscopy, DFT calculations of ground state vibrations (Table S2), time resolved fluorescence studies (Table S3), DADS corresponding to EADS presented above along with quality of fit data, steady state data for Padron/Kohinoor comparison (Table S4). See DOI: https://doi.org/10.1039/d5sc04491j.
