# Supplementary material for: Ultrafast photophysics of a positive reversibly switchable fluorescent protein
Source: Chem Sci. 2025 Aug 7;16(36):16955–69. doi: 10.1039/d5sc04491j (PMC12379806; doi:10.1039/d5sc04491j)
Supplement: SC-016-D5SC04491J-s001 [file SC-016-D5SC04491J-s001.pdf]

Supplementary Information (SI) for Chemical Science.
This journal is © The Royal Society of Chemistry 2025

Full wwPDB X-ray Structure Validation Report (i)

Oct 8, 2024 – 09:41 AM EDT

PDB ID	:	9DVE
Title	:	X-ray crystal structure of Kohinoor reversibly switchable fluorescent protein
Deposited on	:	2024-10-07
Resolution	:	2.15 Å(reported)

This wwPDB validation report is for manuscript review

This is a Full wwPDB X-ray Structure Validation Report.

This report is produced by the wwPDB biocuration pipeline after annotation of the structure.

We welcome your comments at *validation@mail.wwpdb.org*
A user guide is available at
https://www.wwpdb.org/validation/2017/XrayValidationReportHelp
with specific help available everywhere you see the (i) symbol.

The types of validation reports are described at
http://www.wwpdb.org/validation/2017/FAQs#types.

The following versions of software and data (see references (i)) were used in the production of this report:

MolProbity	:	4.02b-467
Mogul	:	2022.3.0, CSD as543be (2022)
Xtriage (Phenix)	:	1.20.1
EDS	:	3.0
Percentile statistics	:	20231227.v01 (using entries in the PDB archive December 27th 2023)
CCP4	:	9.0.003 (Gargrove)
Density-Fitness	:	1.0.11
Ideal geometry (proteins)	:	Engh & Huber (2001)
Ideal geometry (DNA, RNA)	:	Parkinson et al. (1996)
Validation Pipeline (wwPDB-VP)	:	2.39

For Manuscript Review

1 Overall quality at a glance (i)

The following experimental techniques were used to determine the structure:
X-RAY DIFFRACTION

The reported resolution of this entry is 2.15 Å.

Percentile scores (ranging between 0-100) for global validation metrics of the entry are shown in the following graphic. The table shows the number of entries on which the scores are based.

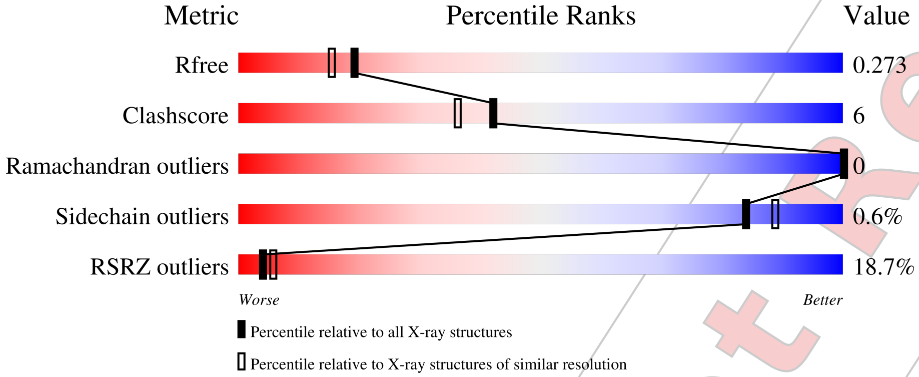

Metric	Whole archive (#Entries)	Similar resolution (#Entries, resolution range(Å))
R_{free}	164625	1881 (2.16-2.16)
Clashscore	180529	2047 (2.16-2.16)
Ramachandran outliers	177936	2027 (2.16-2.16)
Sidechain outliers	177891	2026 (2.16-2.16)
RSRZ outliers	164620	1882 (2.16-2.16)

The table below summarises the geometric issues observed across the polymeric chains and their fit to the electron density. The red, orange, yellow and green segments of the lower bar indicate the fraction of residues that contain outliers for >=3, 2, 1 and 0 types of geometric quality criteria respectively. A grey segment represents the fraction of residues that are not modelled. The numeric value for each fraction is indicated below the corresponding segment, with a dot representing fractions <=5% The upper red bar (where present) indicates the fraction of residues that have poor fit to the electron density. The numeric value is given above the bar.

Mol	Chain	Length	Quality of chain
1	A	225	6% / 85% 10% 5%
1	B	225	12% / 84% 12% •
1	C	225	11% / 88% 6% • 5%
1	D	225	39% / 70% 11% • 18%

For Manuscript Review

2 Entry composition (i)

There are 3 unique types of molecules in this entry. The entry contains 12333 atoms, of which 5791 are hydrogens and 0 are deuteriums.

In the tables below, the ZeroOcc column contains the number of atoms modelled with zero occupancy, the AltConf column contains the number of residues with at least one atom in alternate conformation and the Trace column contains the number of residues modelled with at most 2 atoms.

- Molecule 1 is a protein called Kohinoor.

Mol	Chain	Residues	Atoms	ZeroOcc	AltConf	Trace
1	A	214	Total C H N O S<br>3302 1085 1603 286 318 10	0	0	0
1	B	217	Total C H N O S<br>3236 1073 1553 285 315 10	0	0	0
1	C	213	Total C H N O S<br>3196 1060 1529 283 314 10	0	0	0
1	D	185	Total C H N O S<br>2426 837 1096 227 258 8	0	0	0

- Molecule 2 is DI(HYDROXYETHYL)ETHER (three-letter code: PEG) (formula: $C_4H_{10}O_3$).

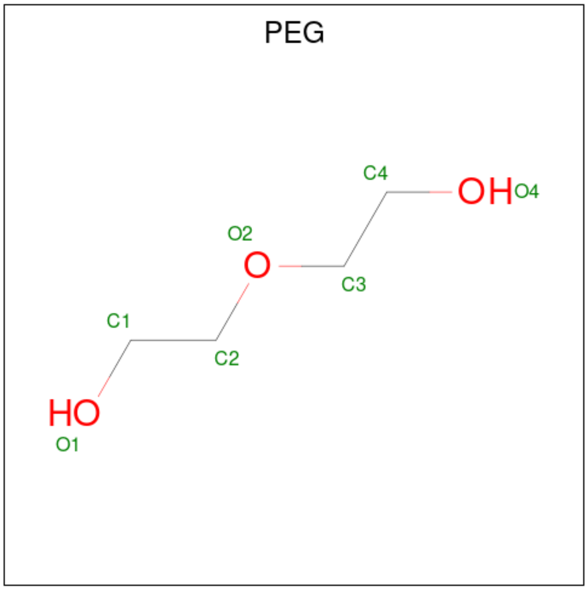

Mol	Chain	Residues	Atoms	ZeroOcc	AltConf
2	A	1	Total C H O<br>17 4 10 3	0	0

- Molecule 3 is water.

For Manuscript Review

Mol	Chain	Residues	Atoms		ZeroOcc	AltConf
3	A	53	Total 53	O 53	0	0
3	B	37	Total 37	O 37	0	0
3	C	44	Total 44	O 44	0	0
3	D	22	Total 22	O 22	0	0

For Manuscript Review

3 Residue-property plots (i)

These plots are drawn for all protein, RNA, DNA and oligosaccharide chains in the entry. The first graphic for a chain summarises the proportions of the various outlier classes displayed in the second graphic. The second graphic shows the sequence view annotated by issues in geometry and electron density. Residues are color-coded according to the number of geometric quality criteria for which they contain at least one outlier: green = 0, yellow = 1, orange = 2 and red = 3 or more. A red dot above a residue indicates a poor fit to the electron density (RSRZ > 2). Stretches of 2 or more consecutive residues without any outlier are shown as a green connector. Residues present in the sample, but not in the model, are shown in grey.

- Molecule 1: Kohinoor

Chain A:
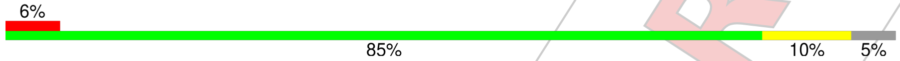

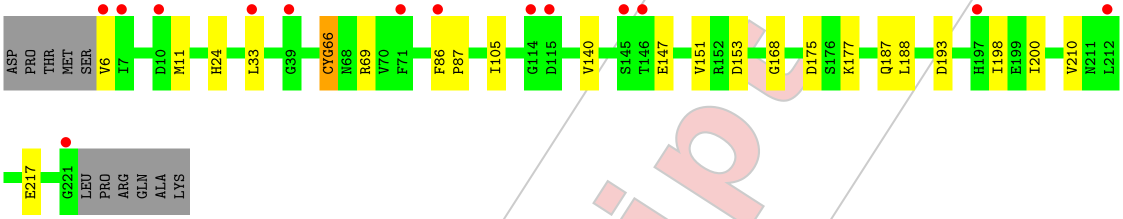

- Molecule 1: Kohinoor

Chain B:
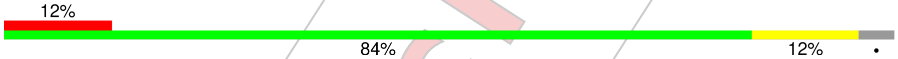

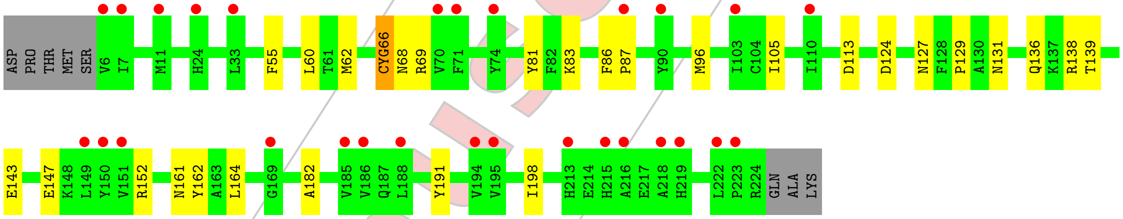

- Molecule 1: Kohinoor

Chain C:
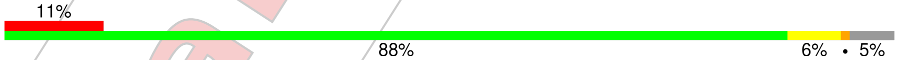

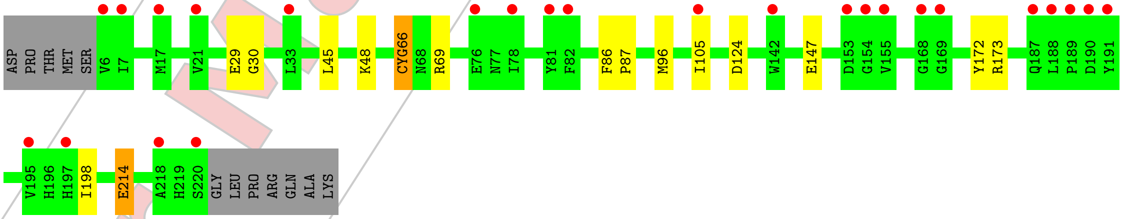

- Molecule 1: Kohinoor

Chain D:
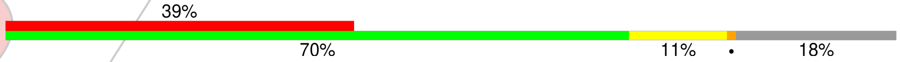

For Manuscript Review

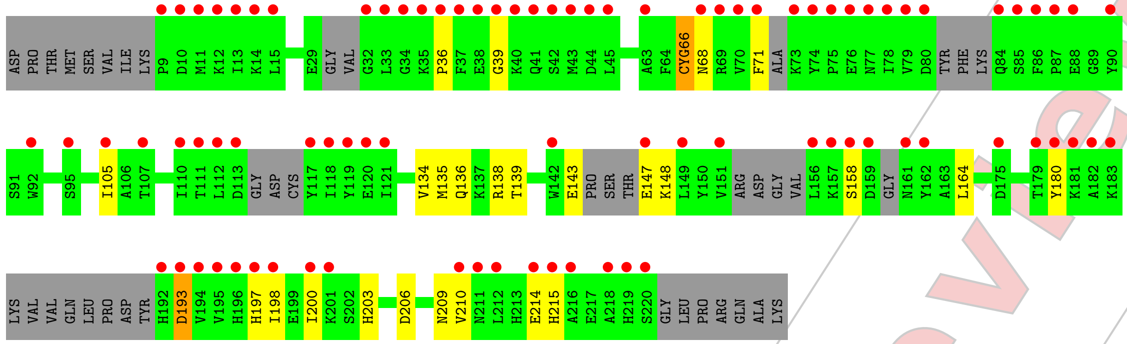

For Manuscript Review

4 Data and refinement statistics (i)

Property	Value	Source
Space group	C 1 2 1	Depositor
Cell constants<br>a, b, c, α, β, γ	138.53Å 73.63Å 93.22Å<br>90.00° 115.86° 90.00°	Depositor
Resolution (Å)	83.89 – 2.15<br>83.89 – 2.15	Depositor<br>EDS
% Data completeness<br>(in resolution range)	95.7 (83.89-2.15)<br>96.0 (83.89-2.15)	Depositor<br>EDS
R_{merge}	0.07	Depositor
R_{sym}	(Not available)	Depositor
$< I/\sigma(I) >$ [1]	1.43 (at 2.14Å)	Xtriage
Refinement program	PHENIX 1.20.1_4487	Depositor
R, R_{free}	0.244 , 0.272<br>0.244 , 0.273	Depositor<br>DCC
R_{free} test set	4036 reflections (4.99%)	wwPDB-VP
Wilson B-factor (Å^2)	50.9	Xtriage
Anisotropy	0.052	Xtriage
Bulk solvent k_{sol}(e/Å^3), B_{sol}(Å^2)	0.42 , 53.6	EDS
L-test for twinning[2]	$< \|L\| >$ = 0.50, $< L^2 >$ = 0.33	Xtriage
Estimated twinning fraction	No twinning to report.	Xtriage
F_o,F_c correlation	0.93	EDS
Total number of atoms	12333	wwPDB-VP
Average B, all atoms (Å^2)	70.0	wwPDB-VP

Xtriage's analysis on translational NCS is as follows: *The largest off-origin peak in the Patterson function is 5.02% of the height of the origin peak. No significant pseudotranslation is detected.*

[1]Intensities estimated from amplitudes.
[2]Theoretical values of $< |L| >$, $< L^2 >$ for acentric reflections are 0.5, 0.333 respectively for untwinned datasets, and 0.375, 0.2 for perfectly twinned datasets.

For Manuscript Review

5 Model quality (i)

5.1 Standard geometry (i)

Bond lengths and bond angles in the following residue types are not validated in this section: PEG, GYC

The Z score for a bond length (or angle) is the number of standard deviations the observed value is removed from the expected value. A bond length (or angle) with $|Z| > 5$ is considered an outlier worth inspection. RMSZ is the root-mean-square of all Z scores of the bond lengths (or angles).

Mol	Chain	Bond lengths		Bond angles	
		RMSZ	#$\|Z\|$ >5	RMSZ	#$\|Z\|$ >5
1	A	0.28	0/1722	0.53	0/2328
1	B	0.27	0/1705	0.53	0/2311
1	C	0.29	0/1690	0.54	0/2291
1	D	0.27	0/1333	0.50	0/1805
All	All	0.28	0/6450	0.53	0/8735

There are no bond length outliers.

There are no bond angle outliers.

There are no chirality outliers.

There are no planarity outliers.

5.2 Too-close contacts (i)

In the following table, the Non-H and H(model) columns list the number of non-hydrogen atoms and hydrogen atoms in the chain respectively. The H(added) column lists the number of hydrogen atoms added and optimized by MolProbity. The Clashes column lists the number of clashes within the asymmetric unit, whereas Symm-Clashes lists symmetry-related clashes.

Mol	Chain	Non-H	H(model)	H(added)	Clashes	Symm-Clashes
1	A	1699	1603	1611	19	0
1	B	1683	1553	1562	26	0
1	C	1667	1529	1536	14	0
1	D	1330	1096	1098	20	0
2	A	7	10	10	0	0
3	A	53	0	0	7	0
3	B	37	0	0	11	0
3	C	44	0	0	6	0
3	D	22	0	0	2	0
All	All	6542	5791	5817	76	0

For Manuscript Review

The all-atom clashscore is defined as the number of clashes found per 1000 atoms (including hydrogen atoms). The all-atom clashscore for this structure is 6.

All (76) close contacts within the same asymmetric unit are listed below, sorted by their clash magnitude.

Atom-1	Atom-2	Interatomic distance (Å)	Clash overlap (Å)
1:A:193:ASP:OD1	3:A:401:HOH:O	1.86	0.92
1:C:96:MET:SD	3:C:343:HOH:O	2.35	0.84
1:B:136:GLN:OE1	1:B:138:ARG:NH1	2.13	0.82
1:B:113:ASP:OD2	3:B:301:HOH:O	1.97	0.81
1:D:206:ASP:OD2	3:D:301:HOH:O	1.98	0.80
1:B:143:GLU:O	3:B:302:HOH:O	1.99	0.79
1:B:161:ASN:ND2	3:B:305:HOH:O	2.13	0.75
1:B:152:ARG:O	3:B:303:HOH:O	2.06	0.73
1:D:143:GLU:OE2	3:D:302:HOH:O	2.10	0.69
1:B:66:GYC:HE2	1:B:198:ILE:HB	1.75	0.69
1:B:129:PRO:O	3:B:304:HOH:O	2.10	0.68
1:D:66:GYC:HE2	1:D:198:ILE:HB	1.77	0.66
1:B:127:ASN:O	3:B:306:HOH:O	2.13	0.65
1:C:66:GYC:HE2	1:C:198:ILE:HB	1.77	0.65
1:C:69:ARG:NH1	1:C:147:GLU:OE1	2.29	0.65
1:A:140:VAL:O	3:A:403:HOH:O	2.13	0.65
1:B:124:ASP:OD1	3:B:307:HOH:O	2.15	0.65
1:A:66:GYC:HE2	1:A:198:ILE:HB	1.78	0.64
1:A:168:GLY:N	3:A:402:HOH:O	1.93	0.61
1:A:24:HIS:NE2	3:A:405:HOH:O	2.24	0.60
1:B:69:ARG:NH1	1:B:147:GLU:OE1	2.33	0.60
1:A:177:LYS:NZ	3:A:404:HOH:O	2.33	0.60
1:A:69:ARG:NH1	1:A:147:GLU:OE1	2.35	0.59
1:C:214:GLU:OE2	3:C:302:HOH:O	2.17	0.59
1:D:66:GYC:N2	1:D:66:GYC:HD2	2.18	0.59
1:B:62:MET:HE1	1:B:162:TYR:CE2	2.38	0.58
1:C:29:GLU:OE1	1:C:48:LYS:NZ	2.35	0.58
1:A:175:ASP:OD2	3:A:404:HOH:O	2.17	0.58
1:B:66:GYC:HD2	1:B:66:GYC:N2	2.19	0.57
1:C:173:ARG:NH2	3:C:304:HOH:O	2.23	0.57
1:A:175:ASP:CG	3:A:404:HOH:O	2.44	0.56
1:C:172:TYR:OH	3:C:303:HOH:O	2.18	0.56
1:C:66:GYC:HD2	1:C:66:GYC:N2	2.23	0.54
1:A:66:GYC:HD2	1:A:66:GYC:N2	2.22	0.54
1:C:173:ARG:NE	3:C:304:HOH:O	2.26	0.53
1:D:66:GYC:N2	1:D:66:GYC:CD2	2.71	0.53
1:A:105:ILE:HG12	1:D:105:ILE:HD12	1.90	0.52

Continued on next page...

For Manuscript Review

Continued from previous page...

Atom-1	Atom-2	Interatomic distance (Å)	Clash overlap (Å)
1:D:197:HIS:O	1:D:214:GLU:HA	2.11	0.52
1:B:131:ASN:OD1	3:B:308:HOH:O	2.19	0.51
1:D:136:GLN:HB2	1:D:138:ARG:HD3	1.93	0.51
1:D:193:ASP:N	1:D:193:ASP:OD1	2.44	0.51
1:D:66:GYC:C3	1:D:68:ASN:N	2.74	0.50
1:B:66:GYC:N2	1:B:66:GYC:CD2	2.73	0.50
1:A:151:VAL:HG11	1:A:188:LEU:HD13	1.92	0.50
1:C:66:GYC:N2	1:C:66:GYC:CD2	2.75	0.49
1:A:153:ASP:O	1:B:191:TYR:CD1	2.65	0.49
1:B:55:PHE:HE1	1:B:60:LEU:HD11	1.77	0.49
1:B:96:MET:SD	3:B:337:HOH:O	2.60	0.49
1:B:62:MET:CE	1:B:162:TYR:CE2	2.97	0.48
1:A:66:GYC:N2	1:A:66:GYC:CD2	2.77	0.47
1:A:11:MET:O	1:A:33:LEU:HD12	2.15	0.47
1:B:81:TYR:OH	1:B:182:ALA:N	2.48	0.47
1:B:86:PHE:HB3	1:B:87:PRO:HA	1.98	0.46
1:C:30:GLY:HA3	1:C:45:LEU:HD23	1.98	0.46
1:C:124:ASP:HB3	3:C:305:HOH:O	2.16	0.45
1:C:86:PHE:HB3	1:C:87:PRO:HA	1.98	0.44
1:D:66:GYC:HB11	1:D:198:ILE:HD12	1.98	0.44
1:D:200:ILE:HG23	1:D:210:VAL:HG13	1.99	0.44
1:B:83:LYS:NZ	3:B:313:HOH:O	2.51	0.43
1:B:105:ILE:HG12	1:C:105:ILE:HD12	2.00	0.43
1:D:134:VAL:HG12	1:D:135:MET:HE2	1.99	0.43
1:A:86:PHE:HB3	1:A:87:PRO:HA	2.01	0.43
1:D:203:HIS:HA	1:D:209:ASN:O	2.17	0.43
1:D:66:GYC:C3	1:D:68:ASN:HB2	2.49	0.43
1:D:147:GLU:OE2	1:D:180:TYR:OH	2.36	0.43
1:D:139:THR:HB	1:D:164:LEU:HD21	2.01	0.42
1:B:139:THR:HB	1:B:164:LEU:HD21	2.02	0.42
1:B:81:TYR:CZ	1:B:182:ALA:HB2	2.54	0.42
1:B:68:ASN:N	3:B:312:HOH:O	2.53	0.42
1:A:6:VAL:O	1:A:6:VAL:HG13	2.20	0.42
1:D:148:LYS:O	1:D:158:SER:HA	2.20	0.41
1:B:55:PHE:CE1	1:B:60:LEU:HD11	2.55	0.41
1:A:6:VAL:O	1:A:6:VAL:HG22	2.21	0.41
1:D:39:GLY:O	1:D:215:HIS:HA	2.20	0.40
1:D:36:PRO:HA	1:D:71:PHE:HA	2.03	0.40
1:A:200:ILE:HG23	1:A:210:VAL:HG13	2.03	0.40

There are no symmetry-related clashes.

For Manuscript Review

5.3 Torsion angles (i)

5.3.1 Protein backbone (i)

In the following table, the Percentiles column shows the percent Ramachandran outliers of the chain as a percentile score with respect to all X-ray entries followed by that with respect to entries of similar resolution.

The Analysed column shows the number of residues for which the backbone conformation was analysed, and the total number of residues.

Mol	Chain	Analysed	Favoured	Allowed	Outliers	Percentiles	
1	A	209/225 (93%)	207 (99%)	2 (1%)	0	100	100
1	B	212/225 (94%)	205 (97%)	7 (3%)	0	100	100
1	C	208/225 (92%)	205 (99%)	3 (1%)	0	100	100
1	D	164/225 (73%)	163 (99%)	1 (1%)	0	100	100
All	All	793/900 (88%)	780 (98%)	13 (2%)	0	100	100

There are no Ramachandran outliers to report.

5.3.2 Protein sidechains (i)

In the following table, the Percentiles column shows the percent sidechain outliers of the chain as a percentile score with respect to all X-ray entries followed by that with respect to entries of similar resolution.

The Analysed column shows the number of residues for which the sidechain conformation was analysed, and the total number of residues.

Mol	Chain	Analysed	Rotameric	Outliers	Percentiles	
1	A	177/192 (92%)	175 (99%)	2 (1%)	70	75
1	B	169/192 (88%)	169 (100%)	0	100	100
1	C	169/192 (88%)	168 (99%)	1 (1%)	84	89
1	D	116/192 (60%)	115 (99%)	1 (1%)	75	81
All	All	631/768 (82%)	627 (99%)	4 (1%)	84	89

All (4) residues with a non-rotameric sidechain are listed below:

Mol	Chain	Res	Type
1	A	187	GLN
1	A	217	GLU

Continued on next page...

For Manuscript Review

Continued from previous page...

Mol	Chain	Res	Type
1	C	214	GLU
1	D	193	ASP

Sometimes sidechains can be flipped to improve hydrogen bonding and reduce clashes. There are no such sidechains identified.

5.3.3 RNA ⓘ

There are no RNA molecules in this entry.

5.4 Non-standard residues in protein, DNA, RNA chains ⓘ

4 non-standard protein/DNA/RNA residues are modelled in this entry.

In the following table, the Counts columns list the number of bonds (or angles) for which Mogul statistics could be retrieved, the number of bonds (or angles) that are observed in the model and the number of bonds (or angles) that are defined in the Chemical Component Dictionary. The Link column lists molecule types, if any, to which the group is linked. The Z score for a bond length (or angle) is the number of standard deviations the observed value is removed from the expected value. A bond length (or angle) with $|Z| > 2$ is considered an outlier worth inspection. RMSZ is the root-mean-square of all Z scores of the bond lengths (or angles).

Mol	Type	Chain	Res	Link	Bond lengths			Bond angles		
					Counts	RMSZ	$\#\|Z\| > 2$	Counts	RMSZ	$\#\|Z\| > 2$
1	GYC	A	66	1	21,22,23	3.12	7 (33%)	26,30,32	7.31	13 (50%)
1	GYC	D	66	1	21,22,23	3.36	6 (28%)	26,30,32	8.18	14 (53%)
1	GYC	C	66	1	21,22,23	3.20	6 (28%)	26,30,32	7.69	14 (53%)
1	GYC	B	66	1	21,22,23	3.23	6 (28%)	26,30,32	7.54	13 (50%)

In the following table, the Chirals column lists the number of chiral outliers, the number of chiral centers analysed, the number of these observed in the model and the number defined in the Chemical Component Dictionary. Similar counts are reported in the Torsion and Rings columns. '-' means no outliers of that kind were identified.

Mol	Type	Chain	Res	Link	Chirals	Torsions	Rings
1	GYC	A	66	1	-	2/10/29/30	0/2/2/2
1	GYC	D	66	1	-	2/10/29/30	0/2/2/2
1	GYC	C	66	1	-	2/10/29/30	0/2/2/2
1	GYC	B	66	1	-	2/10/29/30	0/2/2/2

For Manuscript Review

All (25) bond length outliers are listed below:

Mol	Chain	Res	Type	Atoms	Z	Observed(Å)	Ideal(Å)
1	D	66	GYC	CA2-C2	11.38	1.60	1.48
1	C	66	GYC	CA2-C2	10.33	1.59	1.48
1	B	66	GYC	CA2-C2	10.26	1.59	1.48
1	A	66	GYC	CA2-C2	9.63	1.58	1.48
1	A	66	GYC	C1-N2	6.53	1.41	1.32
1	B	66	GYC	C1-N2	6.39	1.41	1.32
1	C	66	GYC	C1-N2	6.18	1.41	1.32
1	D	66	GYC	C1-N2	5.68	1.40	1.32
1	D	66	GYC	CG2-CB2	5.53	1.57	1.46
1	B	66	GYC	CG2-CB2	4.92	1.55	1.46
1	C	66	GYC	CG2-CB2	4.90	1.55	1.46
1	A	66	GYC	CG2-CB2	4.59	1.55	1.46
1	A	66	GYC	CA2-N2	4.45	1.48	1.38
1	B	66	GYC	CA2-N2	4.31	1.47	1.38
1	C	66	GYC	CA2-N2	4.17	1.47	1.38
1	D	66	GYC	CA2-N2	3.82	1.46	1.38
1	D	66	GYC	C2-N3	3.67	1.48	1.40
1	C	66	GYC	C2-N3	3.55	1.48	1.40
1	B	66	GYC	C2-N3	3.55	1.48	1.40
1	A	66	GYC	C2-N3	3.37	1.47	1.40
1	B	66	GYC	C1-N3	2.29	1.41	1.37
1	A	66	GYC	O2-C2	-2.09	1.18	1.23
1	A	66	GYC	C1-N3	2.09	1.40	1.37
1	C	66	GYC	O2-C2	-2.05	1.18	1.23
1	D	66	GYC	OH-CZ	2.02	1.41	1.37

All (54) bond angle outliers are listed below:

Mol	Chain	Res	Type	Atoms	Z	Observed(^{o})	Ideal(^{o})
1	D	66	GYC	CB2-CA2-C2	30.34	159.13	122.36
1	C	66	GYC	CB2-CA2-C2	28.25	156.60	122.36
1	B	66	GYC	CB2-CA2-C2	28.02	156.32	122.36
1	A	66	GYC	CB2-CA2-C2	26.62	154.62	122.36
1	D	66	GYC	CB2-CA2-N2	-24.16	95.95	128.76
1	C	66	GYC	CB2-CA2-N2	-22.49	98.22	128.76
1	B	66	GYC	CB2-CA2-N2	-22.19	98.63	128.76
1	A	66	GYC	CB2-CA2-N2	-20.94	100.33	128.76
1	A	66	GYC	O2-C2-CA2	-8.34	125.70	131.02
1	C	66	GYC	O2-C2-CA2	-7.76	126.07	131.02
1	B	66	GYC	O2-C2-CA2	-7.07	126.51	131.02
1	C	66	GYC	CA1-C1-N3	-6.62	116.22	124.84
1	A	66	GYC	CA1-C1-N3	-6.60	116.24	124.84

Continued on next page...

For Manuscript Review

Continued from previous page...

Mol	Chain	Res	Type	Atoms	Z	Observed(^{o})	Ideal(^{o})
1	D	66	GYC	CA1-C1-N3	-6.22	116.74	124.84
1	D	66	GYC	CA1-CB1-SG1	-5.94	101.70	114.40
1	B	66	GYC	CA1-C1-N3	-5.88	117.18	124.84
1	D	66	GYC	O2-C2-CA2	-5.81	127.31	131.02
1	D	66	GYC	C2-CA2-N2	-5.64	104.91	108.95
1	A	66	GYC	C2-CA2-N2	-5.45	105.05	108.95
1	B	66	GYC	C2-CA2-N2	-5.44	105.05	108.95
1	C	66	GYC	C2-CA2-N2	-5.27	105.17	108.95
1	D	66	GYC	N3-C1-N2	5.24	115.61	111.48
1	A	66	GYC	CG2-CB2-CA2	5.17	136.01	129.87
1	C	66	GYC	CG2-CB2-CA2	4.93	135.73	129.87
1	D	66	GYC	CG2-CB2-CA2	4.85	135.63	129.87
1	B	66	GYC	CG2-CB2-CA2	4.72	135.47	129.87
1	C	66	GYC	N3-C1-N2	4.35	114.91	111.48
1	A	66	GYC	CA1-CB1-SG1	-3.72	106.45	114.40
1	A	66	GYC	N3-C1-N2	3.45	114.20	111.48
1	C	66	GYC	CA1-CB1-SG1	-3.43	107.07	114.40
1	B	66	GYC	CA1-CB1-SG1	-3.40	107.13	114.40
1	B	66	GYC	N3-C1-N2	3.34	114.12	111.48
1	A	66	GYC	CA1-C1-N2	3.07	129.55	123.57
1	D	66	GYC	C2-N3-C1	-3.06	106.65	108.07
1	C	66	GYC	CD1-CG2-CB2	-2.90	111.25	121.22
1	C	66	GYC	CA1-C1-N2	2.72	128.87	123.57
1	A	66	GYC	CD1-CG2-CB2	-2.67	112.06	121.22
1	B	66	GYC	CA1-C1-N2	2.63	128.70	123.57
1	B	66	GYC	CA2-N2-C1	2.62	107.85	105.80
1	B	66	GYC	CD1-CG2-CB2	-2.52	112.57	121.22
1	A	66	GYC	CA2-C2-N3	2.52	105.61	103.50
1	D	66	GYC	CD1-CG2-CB2	-2.49	112.67	121.22
1	C	66	GYC	CD2-CG2-CB2	2.33	129.24	121.22
1	D	66	GYC	CD2-CG2-CB2	2.30	129.11	121.22
1	D	66	GYC	CA2-N2-C1	2.28	107.58	105.80
1	A	66	GYC	CA2-N2-C1	2.20	107.52	105.80
1	B	66	GYC	CA2-C2-N3	2.16	105.32	103.50
1	C	66	GYC	CA2-C2-N3	2.14	105.30	103.50
1	A	66	GYC	O2-C2-N3	2.10	128.69	124.31
1	D	66	GYC	CA1-C1-N2	2.09	127.65	123.57
1	D	66	GYC	CA2-C2-N3	2.07	105.24	103.50
1	C	66	GYC	O2-C2-N3	2.07	128.63	124.31
1	B	66	GYC	CD2-CG2-CB2	2.06	128.28	121.22
1	C	66	GYC	CA2-N2-C1	2.02	107.38	105.80

There are no chirality outliers.

For Manuscript Review

All (8) torsion outliers are listed below:

Mol	Chain	Res	Type	Atoms
1	A	66	GYC	C3-CA3-N3-C2
1	B	66	GYC	C3-CA3-N3-C2
1	C	66	GYC	C3-CA3-N3-C2
1	D	66	GYC	C3-CA3-N3-C2
1	A	66	GYC	C3-CA3-N3-C1
1	B	66	GYC	C3-CA3-N3-C1
1	C	66	GYC	C3-CA3-N3-C1
1	D	66	GYC	C3-CA3-N3-C1

There are no ring outliers.

4 monomers are involved in 15 short contacts:

Mol	Chain	Res	Type	Clashes	Symm-Clashes
1	A	66	GYC	3	0
1	D	66	GYC	6	0
1	C	66	GYC	3	0
1	B	66	GYC	3	0

5.5 Carbohydrates (i)

There are no oligosaccharides in this entry.

5.6 Ligand geometry (i)

1 ligand is modelled in this entry.

In the following table, the Counts columns list the number of bonds (or angles) for which Mogul statistics could be retrieved, the number of bonds (or angles) that are observed in the model and the number of bonds (or angles) that are defined in the Chemical Component Dictionary. The Link column lists molecule types, if any, to which the group is linked. The Z score for a bond length (or angle) is the number of standard deviations the observed value is removed from the expected value. A bond length (or angle) with $|Z| > 2$ is considered an outlier worth inspection. RMSZ is the root-mean-square of all Z scores of the bond lengths (or angles).

Mol	Type	Chain	Res	Link	Bond lengths			Bond angles		
					Counts	RMSZ	$\#\|Z\| > 2$	Counts	RMSZ	$\#\|Z\| > 2$
2	PEG	A	301	-	6,6,6	0.12	0	5,5,5	0.10	0

In the following table, the Chirals column lists the number of chiral outliers, the number of chiral centers analysed, the number of these observed in the model and the number defined in the Chemical Component Dictionary. Similar counts are reported in the Torsion and Rings columns.

For Manuscript Review

'-' means no outliers of that kind were identified.

Mol	Type	Chain	Res	Link	Chirals	Torsions	Rings
2	PEG	A	301	-	-	1/4/4/4	-

There are no bond length outliers.

There are no bond angle outliers.

There are no chirality outliers.

All (1) torsion outliers are listed below:

Mol	Chain	Res	Type	Atoms
2	A	301	PEG	O2-C3-C4-O4

There are no ring outliers.

No monomer is involved in short contacts.

5.7 Other polymers (i)

There are no such residues in this entry.

5.8 Polymer linkage issues (i)

The following chains have linkage breaks:

Mol	Chain	Number of breaks
1	D	1

All chain breaks are listed below:

Model	Chain	Residue-1	Atom-1	Residue-2	Atom-2	Distance (Å)
1	D	66:GYC	C3	68:ASN	N	2.74

For Manuscript Review

6 Fit of model and data (i)

6.1 Protein, DNA and RNA chains (i)

In the following table, the column labelled '#RSRZ> 2' contains the number (and percentage) of RSRZ outliers, followed by percent RSRZ outliers for the chain as percentile scores relative to all X-ray entries and entries of similar resolution. The OWAB column contains the minimum, median, 95th percentile and maximum values of the occupancy-weighted average B-factor per residue. The column labelled 'Q< 0.9' lists the number of (and percentage) of residues with an average occupancy less than 0.9.

Mol	Chain	Analysed	<RSRZ>	#RSRZ>2			OWAB(Å^2)	Q<0.9
1	A	213/225 (94%)	0.80	14 (6%)	26	31	45, 60, 80, 93	0
1	B	216/225 (96%)	1.14	28 (12%)	9	11	52, 72, 90, 104	0
1	C	212/225 (94%)	0.96	25 (11%)	10	12	47, 63, 89, 112	0
1	D	184/225 (81%)	2.09	87 (47%)	0	1	48, 85, 121, 134	0
All	All	825/900 (91%)	1.22	154 (18%)	4	6	45, 68, 109, 134	0

All (154) RSRZ outliers are listed below:

Mol	Chain	Res	Type	RSRZ
1	C	17	MET	6.8
1	D	38	GLU	6.2
1	D	70	VAL	6.0
1	D	9	PRO	6.0
1	D	74	TYR	5.8
1	D	10	ASP	5.7
1	D	86	PHE	5.4
1	D	69	ARG	5.4
1	D	32	GLY	5.0
1	D	37	PHE	4.9
1	D	11	MET	4.8
1	D	63	ALA	4.8
1	D	78	ILE	4.8
1	D	79	VAL	4.8
1	D	80	ASP	4.6
1	D	212	LEU	4.6
1	D	159	ASP	4.5
1	D	71	PHE	4.4
1	D	85	SER	4.2
1	C	191	TYR	4.1
1	D	180	TYR	4.1

Continued on next page...

For Manuscript Review

Continued from previous page...

Mol	Chain	Res	Type	RSRZ
1	D	45	LEU	4.0
1	D	194	VAL	4.0
1	D	73	LYS	4.0
1	D	192	HIS	4.0
1	D	220	SER	4.0
1	D	156	LEU	4.0
1	B	185	VAL	3.9
1	D	117	TYR	3.9
1	B	7	ILE	3.9
1	D	119	TYR	3.9
1	D	33	LEU	3.8
1	D	87	PRO	3.7
1	D	68	ASN	3.7
1	D	112	LEU	3.7
1	D	151	VAL	3.6
1	D	142	TRP	3.6
1	D	14	LYS	3.6
1	A	86	PHE	3.6
1	C	155	VAL	3.5
1	D	196	HIS	3.5
1	C	78	ILE	3.5
1	D	149	LEU	3.5
1	D	77	ASN	3.5
1	D	162	TYR	3.4
1	D	13	ILE	3.4
1	D	183	LYS	3.4
1	B	6	VAL	3.3
1	D	90	TYR	3.3
1	D	84	GLN	3.2
1	B	110	ILE	3.2
1	D	39	GLY	3.2
1	B	219	HIS	3.2
1	D	40	LYS	3.2
1	D	36	PRO	3.2
1	D	118	ILE	3.2
1	D	44	ASP	3.1
1	D	35	LYS	3.1
1	D	218	ALA	3.1
1	D	181	LYS	3.1
1	D	15	LEU	3.1
1	D	113	ASP	3.0
1	D	147	GLU	3.0

Continued on next page...

For Manuscript Review

Continued from previous page...

Mol	Chain	Res	Type	RSRZ
1	D	215	HIS	3.0
1	A	39	GLY	3.0
1	D	195	VAL	3.0
1	A	6	VAL	2.9
1	B	11	MET	2.9
1	D	76	GLU	2.9
1	D	157	LYS	2.9
1	B	222	LEU	2.8
1	C	21	VAL	2.8
1	C	188	LEU	2.8
1	A	71	PHE	2.8
1	B	103	ILE	2.8
1	D	43	MET	2.8
1	D	193	ASP	2.8
1	D	161	ASN	2.8
1	A	221	GLY	2.8
1	A	10	ASP	2.8
1	D	216	ALA	2.8
1	A	115	ASP	2.8
1	C	190	ASP	2.8
1	D	121	ILE	2.7
1	D	219	HIS	2.7
1	D	158	SER	2.6
1	B	70	VAL	2.6
1	B	24	HIS	2.6
1	B	215	HIS	2.6
1	B	33	LEU	2.6
1	B	74	TYR	2.6
1	B	151	VAL	2.6
1	B	169	GLY	2.6
1	D	214	GLU	2.6
1	A	146	THR	2.6
1	B	71	PHE	2.6
1	A	145	SER	2.6
1	C	81	TYR	2.6
1	B	87	PRO	2.6
1	D	75	PRO	2.6
1	C	220	SER	2.6
1	D	42	SER	2.6
1	C	6	VAL	2.5
1	C	168	GLY	2.5
1	D	111	THR	2.5

Continued on next page...

For Manuscript Review

Continued from previous page...

Mol	Chain	Res	Type	RSRZ
1	B	188	LEU	2.5
1	C	153	ASP	2.5
1	D	200	ILE	2.5
1	D	110	ILE	2.4
1	B	223	PRO	2.4
1	C	76	GLU	2.4
1	D	211	ASN	2.4
1	D	107	THR	2.4
1	C	189	PRO	2.3
1	D	95	SER	2.3
1	D	198	ILE	2.3
1	D	201	LYS	2.3
1	B	213	HIS	2.3
1	D	182	ALA	2.3
1	B	194	VAL	2.3
1	D	41	GLN	2.3
1	A	114	GLY	2.3
1	C	154	GLY	2.3
1	D	88	GLU	2.3
1	C	7	ILE	2.3
1	D	105	ILE	2.3
1	A	7	ILE	2.3
1	C	218	ALA	2.3
1	A	212	LEU	2.2
1	C	195	VAL	2.2
1	D	210	VAL	2.2
1	C	142	TRP	2.2
1	B	186	VAL	2.2
1	D	197	HIS	2.2
1	B	218	ALA	2.2
1	D	175	ASP	2.2
1	C	82	PHE	2.1
1	D	179	THR	2.1
1	B	150	TYR	2.1
1	A	197	HIS	2.1
1	C	169	GLY	2.1
1	D	34	GLY	2.1
1	B	149	LEU	2.1
1	C	105	ILE	2.1
1	D	120	GLU	2.1
1	B	216	ALA	2.1
1	B	90	TYR	2.0

Continued on next page...

For Manuscript Review

Continued from previous page...

Mol	Chain	Res	Type	RSRZ
1	B	195	VAL	2.0
1	C	197	HIS	2.0
1	A	33	LEU	2.0
1	C	33	LEU	2.0
1	C	187	GLN	2.0
1	D	12	LYS	2.0
1	D	92	TRP	2.0

6.2 Non-standard residues in protein, DNA, RNA chains (i)

In the following table, the Atoms column lists the number of modelled atoms in the group and the number defined in the chemical component dictionary. The B-factors column lists the minimum, median, 95th percentile and maximum values of B factors of atoms in the group. The column labelled 'Q< 0.9' lists the number of atoms with occupancy less than 0.9.

Mol	Type	Chain	Res	Atoms	RSCC	RSR	B-factors(Å^2)	Q<0.9
1	GYC	C	66	21/22	0.85	0.17	44,54,61,95	0
1	GYC	B	66	21/22	0.88	0.17	56,63,70,75	0
1	GYC	A	66	21/22	0.89	0.16	44,51,65,70	0
1	GYC	D	66	21/22	0.91	0.16	75,81,86,98	0

6.3 Carbohydrates (i)

There are no monosaccharides in this entry.

6.4 Ligands (i)

In the following table, the Atoms column lists the number of modelled atoms in the group and the number defined in the chemical component dictionary. The B-factors column lists the minimum, median, 95th percentile and maximum values of B factors of atoms in the group. The column labelled 'Q< 0.9' lists the number of atoms with occupancy less than 0.9.

Mol	Type	Chain	Res	Atoms	RSCC	RSR	B-factors(Å^2)	Q<0.9
2	PEG	A	301	7/7	0.91	0.10	56,68,72,79	0

6.5 Other polymers (i)

There are no such residues in this entry.

For Manuscript Review
