# Supplementary material for: Ultrafast photophysics of a positive reversibly switchable fluorescent protein
Source: Chem Sci. 2025 Aug 7;16(36):16955–69. doi: 10.1039/d5sc04491j (PMC12379806; doi:10.1039/d5sc04491j)
Supplement: SC-016-D5SC04491J-s002 [file SC-016-D5SC04491J-s002.pdf]

Supplementary Information (SI) for Chemical Science.
This journal is © The Royal Society of Chemistry 2025

Ultrafast Photophysics of a Positive Reversibly Switchable Fluorescent Protein

Anam Fatima,[1] YongLe He,[2] James N. Iuliano,[2] Gregory M. Greetham,[3] Partha Malakar,[3] Christopher Hall,[1,6] Helena A. Woroniecka,[2] Brian C. Richardson,[5] Jarrod B. French,[5]Andras Lukacs,[4*] Peter J. Tonge[2*] and Stephen R. Meech[1*]

1. School of Chemistry, University of East Anglia, Norwich NR4 7TJ, U.K; 2. Department of Chemistry, Stony Brook University, Stony Brook, New York 11794, United States; 3 Central Laser Facility, Research Complex at Harwell, Rutherford Appleton Laboratory, Didcot OX11 0QX, U.K. 4. Department of Biophysics, Medical School, University of Pecs, 7624 Pecs, Hungary. 5. 5. The Hormel Institute, University of Minnesota, Austin MN, 55912, United States. 6. School of Chemistry, University of Melbourne, Parkville, VIC, 3010 Australia

*Author for correspondence: s.meech@uea.ac.uk; peter.tonge@stonybrook.edu; Andras.lukacs@aok.pte.hu

Contents

1. Experimental section 3

a.) Steady-State Absorption and Emission 3

b.) Fluorescence up-conversion 3

c.) Visible transient absorption (TA)....................3

d.) **Time-resolved Infra-red spectroscopy (TRIR)**....................4

e.) Time-resolved Multiprobe spectroscopy (TRMPS) 4

f.) Expression and Purification of Kohinoor....................4

g.) Crystallization and Data Collection....................5

h.) **Structure Determination and Refinement** 5

Table S1. Data collection and refinement statistics....................6

i) DFT calculations 7

2. Data Analysis: Tables and Figures 8

Fig S1 Excitation spectra observed at 530 and 450 nm normalized to the respective absorption bands showing 465 nm emission originates from the excitation of the cisH state, and the 530 nm emission arises exclusively from the excitation of the cis− state, indicating no contribution from ESPT. 8

Fig S2 Emission spectra recorded after excitation of the ON and OFF states at 480 nm reveal residual emission (weaker than ON state by a factor of ca.20) from the ON state when the OFF state is excited. 8

Fig S3 (a) Decay associated difference spectra (DADS) retrieved from the global analysis of the OFF-state (trans$^-$) TA data assuming a parallel decay model showing a fast relaxation on the red side of the SE leading to a narrower slightly blue shifted band which then relaxes on a picosecond timescale to yield a 'constant' and (b) Global biexponential fit (plus a long-lived component) of kinetic traces presented at selected wavelengths. The quality of fit is the same for sequential (EADS) and parallel (DADS) models....................9

Fig S4 DFT-calculated infrared transition wavenumbers and intensities of the cis and trans anion forms of the chromophore (uncorrected calculation for gas phase, isolated chromophore). 9

Table S2: The assignments of the vibrational modes of cis^- and $trans^-$ calculated by DFT 10

Fig S5 (a) Decay associated difference spectra (DADS) retrieved from the global analysis of the OFF-state ($trans^-$) of TRIR data assuming a parallel decay model showing a rather complex and wavelength dependent evolution. The intermediate formed in 1.2 ps relaxes to repopulate the ground state (e.g. filling the chromophore bleach signals at 1492, 1587 and 1666 cm^{-1}) in 4.5 ps., which then relaxes on a tens of picoseconds timescale to reform the original state ($trans^-$) and (b) Global biexponential fit (plus a long-lived component) of kinetic traces presented at selected wavelengths. 11

Fig S6 TRIR spectra of the OFF state ($trans^-$) of Kohinoor beyond 40 ps showing continued relaxation to yield a very weak but reproducible signals at 1460 and 1650 cm^{-1} suggesting that these small residual perturbations must arise from the *ca* 1% yield of the cis^- product. 11

Fig S7 (a) Steady state absorption spectra of the ON and OFF states of Kohinoor at pH10 showing the barely resolved cisH band at 380 nm, (b) Emission spectra recorded at 400 and 480 nm excitations and (c) excitation spectra observed at 530 nm emission wavelength. The absence of 450 nm emission band (400 nm excitation) and 380 nm band in excitation spectra strongly indicate lack of ESPT. 12

Fig S8 Time-resolved fluorescence (exc = 400 nm) of ON-state (cisH) of Kohinoor recorded at 460 and 510 nm in water. The sample were measured in a flow cell system and the reservoir was continuously irradiated with 505 nm LED to retain the ON-state. 12

Table S3 Time-constants (τ) and amplitudes (α) obtained by fitting the TRF decay traces using a three sum of exponential (for 510 nm) and a two sum of exponential (for 460 nm) function. τ_{av} denotes the average time of decay. 12

Fig S9 (a) Decay associated difference spectra (DADS) retrieved from the global analysis of the ON-state (cisH) of TA data assuming a parallel decay model showing that the SE of the initial state narrows along with the decay of ESA bands to form an intermediate state in 3 ps, the latter partly refills the ground state in 22 ps and partly decays to form a long lived component 'constant' attributed to minor cis^- population absorbing at the 400 nm and (b) Global biexponential fit (plus a long-lived component) of kinetic traces presented at selected wavelengths. 13

Fig S10 Global biexponential fit (plus a long-lived component) of kinetic traces of TRIR data of ON-state (cisH) of Kohinoor presented at selected wavelengths. 13

Table S4: Comparison of kinetic parameters between Padron0.9[6] and Kohinoor 14

Fig S11 Time-resolved multiprobe IR spectra revealing a long-lived, low-amplitude component characterized by a bleach/transient pair at 1689/1672 cm^{-1}, which is attributed to transH formation. 14

Fig S12 (a) Decay associated difference spectra (DADS) retrieved from the global analysis of the ON-state (cis^-) TRIR data assuming a parallel decay model showing a fast decay of the bleach/transient pair at 1460/1620 cm^{-1} along with a bleach growing at 1578 cm^{-1} that decays on long time-scale, suggesting two chromophore populations with fast and slow excited state decay times and (b) Global monoexponential fit (plus a long-lived component) of kinetic traces presented at selected wavelengths. 15

Fig S13 The pH dependence of the on-state absorption showing that cisH and cis^- are in equilibrium with one another. 15

Fig S14 The structure of Kohinoor compared with Padron0.9 (Figure 2a) focused on the region of the phenolic group. The modest changes in structure suggest small changes in the S145 to phenolic OH distances. Bottom. To highlight changes in the chromophore, the comparison in Figure 2a (chain aligned structures) is made with the two chromophores here aligned by overlapping their phenol rings, highlighting structure changes in and around the chromophore..16

1. Experimental section

a.) Steady-State Absorption and Emission

The UV–Visible ground-state absorption spectra were recorded with a Perkin Elmer Lambda XLS spectrophotometer. Quartz cells of 1 cm optical path were used, and the absorbance was kept below 1 (<50 µM). Emission spectra at room temperature were recorded using Edinburgh Instruments FS5 spectrofluorometer under right angle configuration with a bandwidth of 2 nm in both excitation and emission.

b.) Fluorescence up-conversion

The ultrafast fluorescence up-conversion experiment has been described in detail elsewhere[1] and uses a femtosecond oscillator (Coherent Micra-10), a mode-locked Ti:Sapphire Laser that generates pulses centred at 800 nm with a duration of approximately 20 fs, a repetition rate of 76 MHz, and a power output of around 750 mW. Excitation is by the second harmonic at *ca.* 400 nm and the power at the sample is up to 9 mW (average power). For protein samples, the power was reduced to 2 mW. Up-conversion was performed in a 300-micron BBO crystal, achieving a time resolution of 75 fs, as determined by up-conversion of solvent Raman scattering. To maintain the samples in their original ON- or OFF-states, the reservoir was continuously illuminated with 405 or 505 nm LED depending on the state studied.

c.) Visible transient absorption (TA)

The transient absorption setup used here has been detailed elsewhere[2]. The pump and probe beams were generated using an 800 nm fundamental output beam produced by a Spectra Physics-Mai Tai Laser oscillator and amplified by a Ti:Sapphire regenerative amplifier (Spectra Physics-Spitfire ACE). This amplified output pulse, characterized by a duration of 100 fs at 800 nm, a repetition rate of 1 kHz, and an energy of 5 mJ per pulse, served to drive the optical parametric amplifier (OPA, Light Conversion TOPAS Prime), thereby producing a tuneable pump pulse for sample excitation. A white light continuum (WLC) probe was generated by focusing part of the fundamental 800 nm beam onto a 3 mm thick sapphire window, yielding a broadband continuum ranging from 450 to 800 nm. Transient absorption spectra presented in this work were recorded following sample excitation at 400 or 465 nm. The energy of the pump beam at the sample cell was attenuated to 200 µW (200 nJ). All measurements were conducted in 1 mm flow cuvettes with OD <1 (<concentration 50 µM). To

maintain the samples in their original ON- or OFF-states, the reservoir was continuously illuminated with 405 nm or 505 nm LEDs, respectively.

d.) Time-resolved Infra-red spectroscopy (TRIR)

Time-resolved infrared (TRIR) spectroscopy was conducted using the ULTRA or LifeTime systems at the Central Laser Facility within the Research Complex at Harwell[3]. Transmitted light from the sample was measured with pump-on and pump-off, employing a visible pulse to excite the sample, followed by an infrared probe to capture the vibrational spectrum. The sample was excited with 380 or 475 nm light pulses (200 nJ) in a 150 micron spot size at a repetition rate of 1 kHz, yielding high signal-to-noise spectra with approximately 100 fs resolution. Data were recorded at magic angle polarization conditions. Protein samples, prepared at a concentration of 1–2 mM in D_2O, were analyzed using a 50 µm path length CaF_2 cell. To reduce photobleaching and sample degradation, the sample cell was rastered while the samples were flowed through the cell at a rate of approximately 1.5 mL/min. The spectra were calibrated against the IR spectrum of polystyrene. To maintain the samples in their original ON- or OFF-states, the reservoir was continuously illuminated with 405 nm or 505 nm LEDs, respectively.

e.) Time-resolved Multiprobe spectroscopy (TRMPS)

TRMPS spectra were obtained from 100 fs to 200 µs at the STFC Central Laser Facility. The TRMPS method has been described previously[4], and used by us to study the photoswitching in Kohinoor ON-state (cisH). This method enables time resolved IR spectra to be acquired over 10 decades of time from sub-ps to ms. The sample was analysed using a flow cell, and data were acquired using a 380 nm pump operated at 0.6-0.8 µJ per pulse and a repetition rate of 1 kHz. After the measurements were recorded, the extent of photoconversion was shown to be negligible using absorbance spectroscopy. The spectral resolution was 3 cm^{-1} and the temporal resolution was 200 fs. The spot size was 150 micron and magic angle conditions were used. A typical measurement was acquired during 45 min of data collection. All samples were prepared at 1-2 mM concentration in D_2O buffer. Spectra were calibrated relative to the IR transmission of a pure polystyrene standard sample placed at the sample position.

f.) Expression and Purification of Kohinoor

The pRSETb plasmid that encoded the gene of Kohinoor with the N-terminal hexahistidine-tag was a gift from Takeharu Nagai (Addgene plasmid # 67770). The *E.Coli* Bl21 (DE3) competent cells were transformed with the pRSetb Kohinoor plasmid and plated on an LB-Agar plate containing 100 μg/mL of ampicillin. The overnight culture was prepared by inoculating LB Miller broth, containing 100 μg/mL of ampicillin, with a single colony and incubating at 37 °C in an orbital shaker (250 rpm). After overnight incubation at 37 °C, 10 mL of the overnight culture was inoculated into 1 L of 2x-YT media

(Fisher Bioreagents, BP9743-5) with the appropriate antibiotic. The cultures were incubated at 37 °C in an orbital shaker (250 rpm) until the OD_{600} ~0.6-0.8 was obtained, and then, the temperature was lowered to 18 °C. Once the OD_{600} was ~0.8-1, 1 mM isopropyl β-D-1-thiogalactopyranoside (IPTG, Gold Biosciences) was added to induce protein expression. The cells were harvested after 16 h post-induction by centrifugation at 5,000 RPM (6,238 x g) (4 °C) for 20 min and the cell pellet was stored at −20 °C until needed

The cell pellet was resuspended in 40 ml of lysis buffer (20 mM Tris pH 8, 150 mM NaCl, buffer A) and lysed by sonication. The cell debris was removed by ultracentrifugation at 40,000 rpm (185,511 x g) for 1 h (4 °C) and filtered by a 40μm constrainer (Wards Sciences). The supernatant was then loaded into a 5 ml Ni-NTA column (GE) and washed with 10-20 CV of buffer A containing 10-30 mM of Imidazole. A gradient of 30-500 mM of Imidazole elution was performed, and the protein was eluted at 500 mM Imidazole. Fractions containing protein were pooled and concentrated by a 10 kDa cut-off concentrator and desalted using the HiPrep 26/10 desalting column (GE) with buffer A. (AKTA FPLC purifier). Pure protein fractions were collected, and the purity of the protein was confirmed by SDS-PAGE. The concentration of Kohinoor was determined by the absorbance spectrum using the extinction coefficient (ε_{495} = 62,900 M^{-1} cm^{-1})[5]. The protein was lyophilized and resuspended in D_2O for the TRIR and TRMPS measurements.

g.) Crystallization and Data Collection

For crystal trials, hexahistidine-tagged Kohinoor was used, without removal of the tag, at ~20 mg/mL in buffer A. Sparse matrix screening (Peg/Ion HR2-922, Hampton) using the hanging drop vapor diffusion method was employed to identify the initial crystallization conditions for Kohinoor. The hanging drop consisted of 1 µL of well reservoir and 2 µL of Kohinoor. The trays were incubated at 25 °C for a week before plane-shaped crystals of Kohinoor appeared in a condition containing 0.12 - 0.14 M Magnesium nitrate hexahydrate and 18 to 24% w/v polyethylene glycol 3350. Crystals were soaked in 10% glycerol under the 505 nm LED illumination for ~3 minutes prior to freezing in liquid nitrogen. Data was collected at 100 K at 17-ID-1 (AMX) at the National Synchrotron Light Source II (NSLSII) on an Eiger 9 M detector. The data collection statistics are provided in Table S1.

h.) Structure Determination and Refinement

The data were indexed, integrated, and scaled using XDS and Aimless to a resolution cut-off of 2.15Å. The Kohinoor ON state structure was initially determined by molecular replacement using phenix.phaser-MR with Padron (PDB entry3ZUJ, 97.2% sequence identity) as a search model. Following molecular replacement, the structure including the modified chromophore GYC was refined via iterations of phenix.refine and manual correction in Coot to a final Rfree of 0.2722

Excellent density was observed for chains A and C; density for chains B and D limited the degree to which they could be modelled accurately, which is reflected in the model statistics. As such, only chains A and C were considered in analysis. The data refinement statistics are provided in Table S1.

Table S1. Data collection and refinement statistics.

	Kohinoor
Wavelength	0.9792
Resolution range	83.89 - 2.15 (2.227 - 2.15)
Space group	C 1 2 1
Unit cell	138.53 73.628 93.219 90 115.857 90
Total reflections	128772 (12645)
Unique reflections	44246 (4320)
Multiplicity	2.9 (2.9)
Completeness (%)	96.04 (94.55)
Mean I/sigma(I)	8.05 (1.54)
Wilson B-factor	50.91
R-merge	0.07103 (0.6648)
R-meas	0.08697 (0.8161)
R-pim	0.04954 (0.4679)
CC1/2	0.995 (0.804)
CC*	0.999 (0.944)
Reflections used in refinement	44242 (4100)
Reflections used for R-free	2209 (220)
R-work	0.2440 (0.3695)
R-free	0.2723 (0.4055)
CC(work)	0.921 (0.194)
CC(free)	0.923 (0.068)
Number of non-hydrogen atoms	6542
macromolecules	6295
ligands	101
solvent	156
Protein residues	825
RMS(bonds)	0.003
RMS(angles)	1.10

Ramachandran favored (%)	98.36
Ramachandran allowed (%)	1.64
Ramachandran outliers (%)	0.00
Rotamer outliers (%)	0.63
Clashscore	7.38
Average B-factor	64.43
macromolecules	64.65
ligands	63.86
solvent	55.89
Number of TLS groups	24

Statistics for the highest-resolution shell are shown in parentheses. Table generated by phenix.table_one

i) DFT calculations

We employ DFT at the B3LYP/6-31G* level of theory, using Gaussian16 for calculation of vibrational frequencies and mode assignments. All structures were minimized (no negative frequencies). Gas phase was used rather than any polarizable continuum, since this cannot represent the specific interactions which play roles in proteins.

2. Data Analysis: Tables and Figures

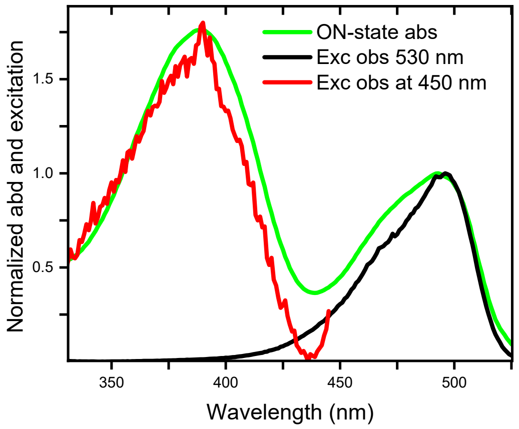

Fig S1 Excitation spectra observed at 530 and 450 nm normalized to the respective absorption bands showing 465 nm emission originates from the excitation of the cisH state, and the 530 nm emission arises exclusively from the excitation of the cis− state, indicating no contribution from ESPT.

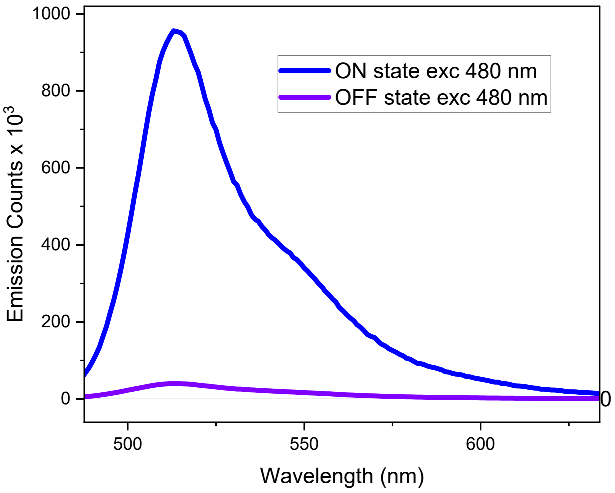

Fig S2 Emission spectra recorded after excitation of the ON and OFF states at 480 nm reveal residual emission (weaker than ON state by a factor of ca.20) from the ON state when the OFF state is excited.

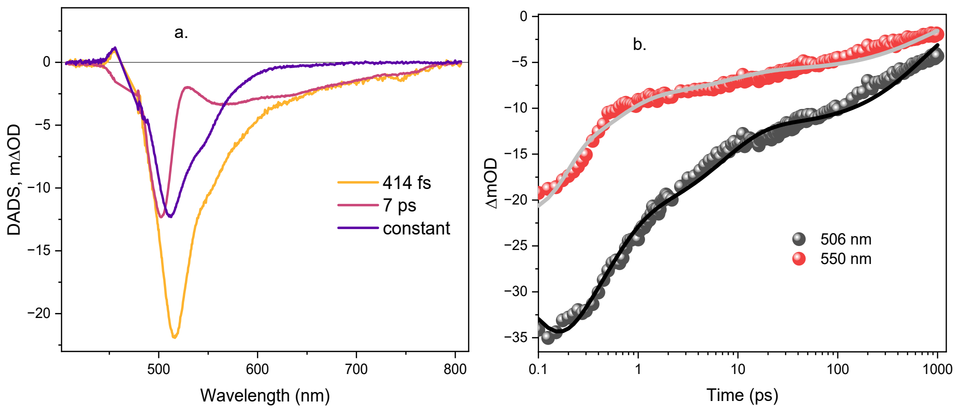

Fig S3 (a) Decay associated difference spectra (DADS) retrieved from the global analysis of the OFF-state (trans⁻) TA data assuming a parallel decay model showing a fast relaxation on the red side of the SE leading to a narrower slightly blue shifted band which then relaxes on a picosecond timescale to yield a 'constant' and (b) Global biexponential fit (plus a long-lived component) of kinetic traces presented at selected wavelengths. The quality of fit is the same for sequential (EADS) and parallel (DADS) models.

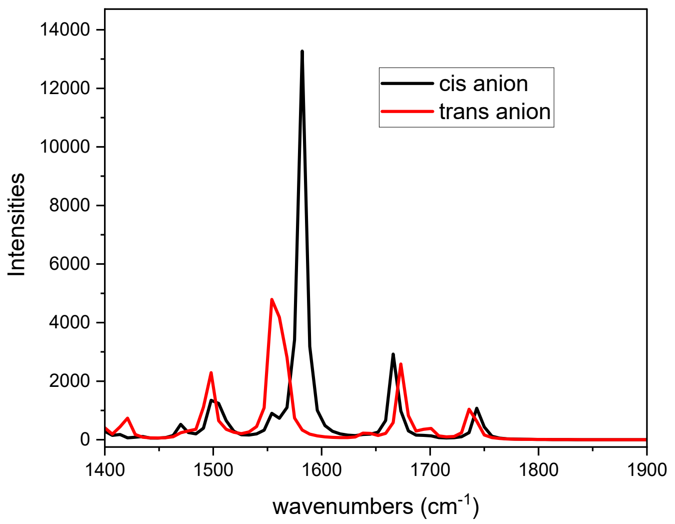

Fig S4 DFT-calculated infrared transition wavenumbers and intensities of the cis and trans anion forms of the chromophore (uncorrected calculation for gas phase, isolated chromophore).

Table S2: The assignments of the vibrational modes of cis$^-$ and trans$^-$ calculated by DFT

Cis$^-$ /cm^{-1}	Trans$^-$ /cm^{-1}	assignment[a]	Exp (cis$^-$, DMSO)[b] /cm^{-1}
1669	1663	C=O	1665(w)
1624(vw)	1624(w)		
1595	1606	C=C-C=N	1630
1513	1497/1489	Ph+C=C	1570
1488	1450	Ph+C=C	
1441	1431	Ph (dl)	1500

All calculated wavenumbers are multiplied by the recommended correction factor 0.957 for comparison to experiment[a] assignment based on major DFT displacements – all modes are to a greater or lesser extent delocalised over the entire molecule (where this is especially marked then marked dl) [b] Experiment in DMSO – the four signals observed are assigned to the most intense calculated modes. Abbreviations vw – very weak; w – weak.

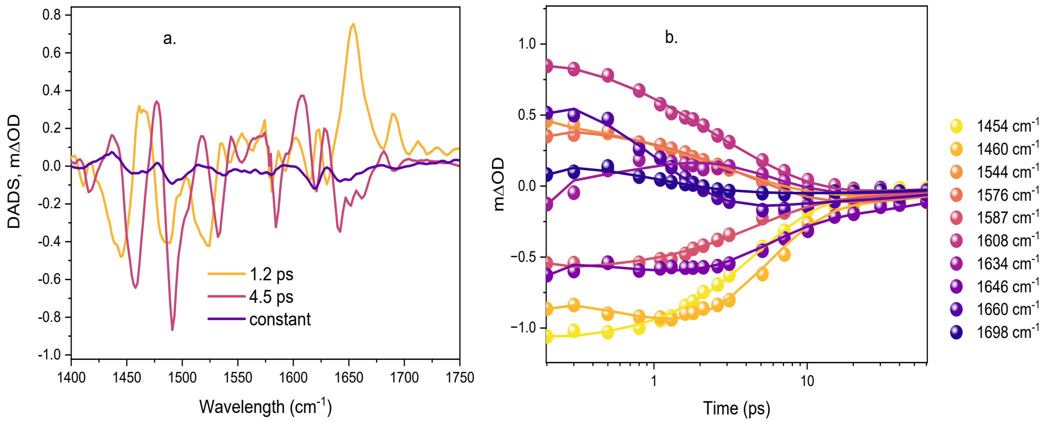

Fig S5 (a) Decay associated difference spectra (DADS) retrieved from the global analysis of the OFF-state (trans$^-$) of TRIR data assuming a parallel decay model showing a rather complex and wavelength dependent evolution. The intermediate formed in 1.2 ps relaxes to repopulate the ground state (e.g. filling the chromophore bleach signals at 1492, 1587 and 1666 cm^{-1}) in 4.5 ps., which then relaxes on a tens of picoseconds timescale to reform the original state (trans$^-$) and (b) Global biexponential fit (plus a long-lived component) of kinetic traces presented at selected wavelengths.

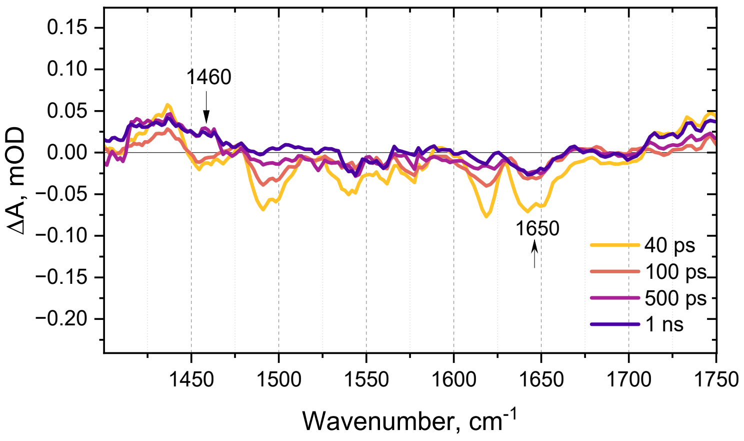

Fig S6 TRIR spectra of the OFF state (trans⁻) of Kohinoor beyond 40 ps showing continued relaxation to yield a very weak but reproducible signals at 1460 and 1650 cm^{-1} suggesting that these small residual perturbations must arise from the *ca* 1% yield of the cis⁻ product.

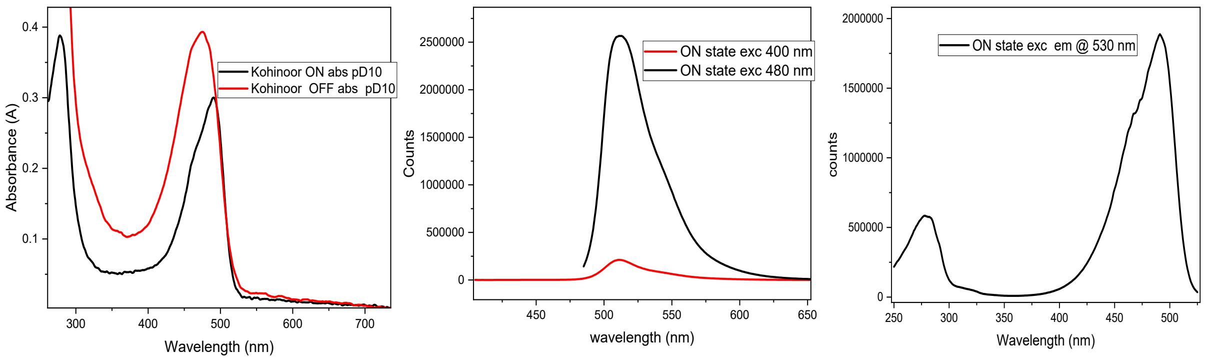

Fig S7 (a) Steady state absorption spectra of the ON and OFF states of Kohinoor at pH10 showing the barely resolved cisH band at 380 nm, (b) Emission spectra recorded at 400 and 480 nm excitations and (c) excitation spectra observed at 530 nm emission wavelength. The absence of 450 nm emission band (400 nm excitation) and 380 nm band in excitation spectra strongly indicate lack of ESPT.

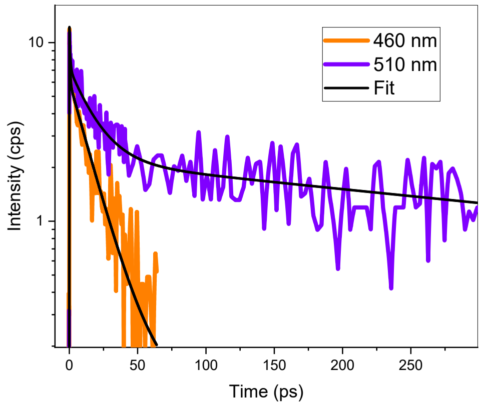

Fig S8 Time-resolved fluorescence (exc = 400 nm) of ON-state (cisH) of Kohinoor recorded at 460 and 510 nm in water. The sample were measured in a flow cell system and the reservoir was continuously irradiated with 505 nm LED to retain the ON-state.

Table S3 Time-constants (τ) and amplitudes (α) obtained by fitting the TRF decay traces using a three sum of exponential (for 510 nm) and a two sum of exponential (for 460 nm) function. τ_{av} denotes the average time of decay.

	α_1	τ_1 (ps)	α_2	τ_2 (ps)	α_3	τ_3 (ps)	τ_{av} (ps)
510 nm	3.3	1.2	4.7	20.2	2	666	143
460 nm	7.2	0.38	6.3	9.1	--	--	4.4

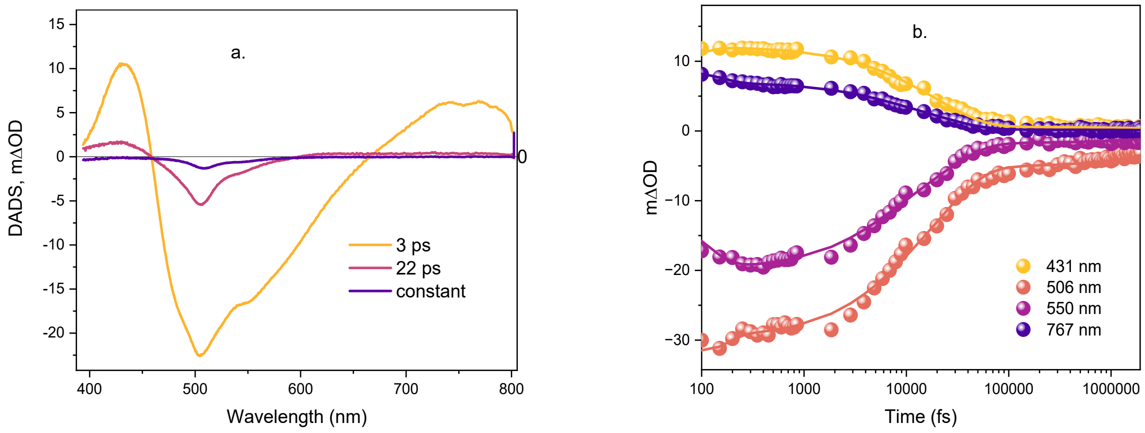

Fig S9 (a) Decay associated difference spectra (DADS) retrieved from the global analysis of the ON-state (cisH) of TA data assuming a parallel decay model showing that the SE of the initial state narrows along with the decay of ESA bands to form an intermediate state in 3 ps, the latter partly refills the ground state in 22 ps and partly decays to form a long lived component 'constant' attributed to minor cis^{-} population absorbing at the 400 nm and (b) Global biexponential fit (plus a long-lived component) of kinetic traces presented at selected wavelengths.

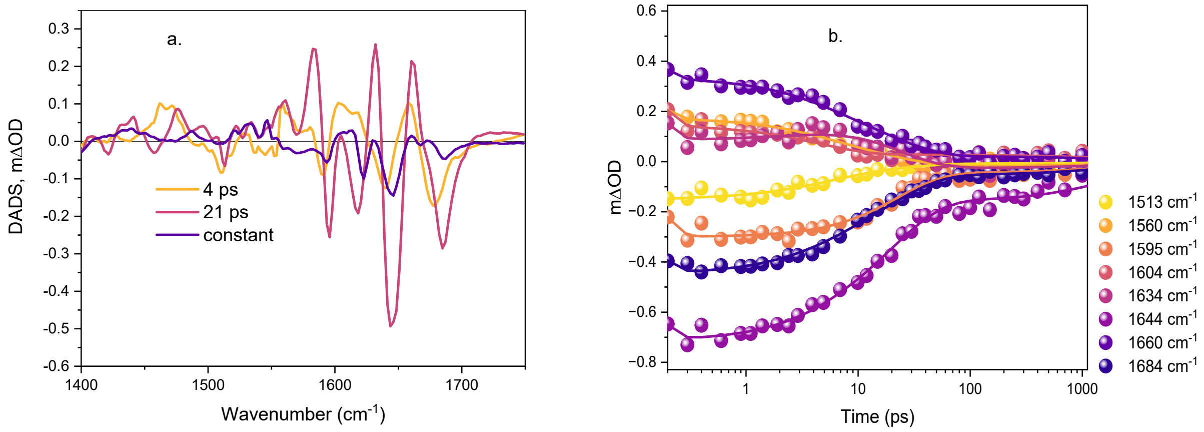

Fig S10 Global biexponential fit (plus a long-lived component) of kinetic traces of TRIR data of ON-state (cisH) of Kohinoor presented at selected wavelengths.

Table S4: Comparison of kinetic parameters between Padron0.9[6] and Kohinoor

	**Relaxation times (exc 400 nm)**	**Relaxation times (exc 500 nm)**	**Quantum yields**	**pKa**
Padron0.9	1 ps, 6.5 ps, and 1 ns	5 ps and 1.2 ns	$\Phi_{off\ to\ on}$ 0.3%<br>$\Phi_{on\ to\ off}$ 2%	10
Kohinoor	3 ps, 22 ps, 3 ns	400 fs, 7 ps and 3 ns	$\Phi_{off\ to\ on}$ 1%<br>$\Phi_{on\ to\ off}$ 8%	8.5

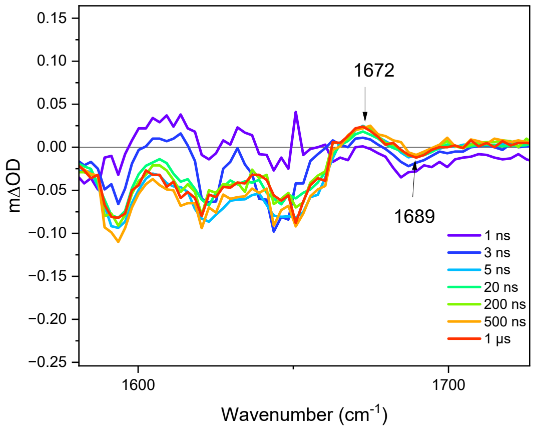

Fig S11 Time-resolved multiprobe IR spectra revealing a long-lived, low-amplitude component characterized by a bleach/transient pair at 1689/1672 cm^{-1}, which is attributed to transH formation.

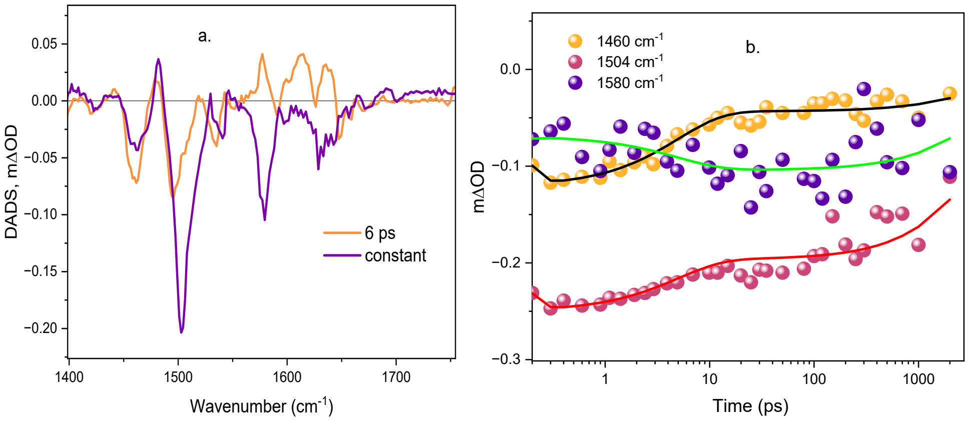

Fig S12 (a) Decay associated difference spectra (DADS) retrieved from the global analysis of the ON-state (cis⁻) TRIR data assuming a parallel decay model showing a fast decay of the bleach/transient pair at 1460/1620 cm^{-1} along with a bleach growing at 1578 cm^{-1} that decays on long time-scale, suggesting two chromophore populations with fast and slow excited state decay times and (b) Global monoexponential fit (plus a long-lived component) of kinetic traces presented at selected wavelengths.

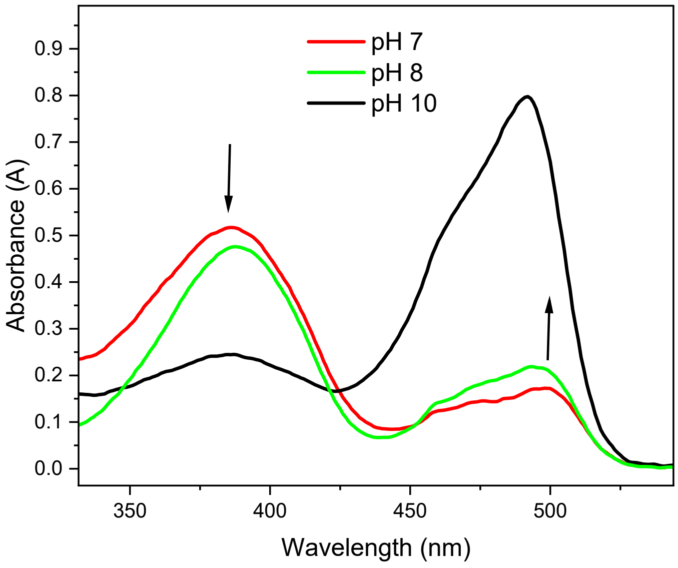

Fig S13 The pH dependence of the on-state absorption showing that cisH and cis⁻ are in equilibrium.

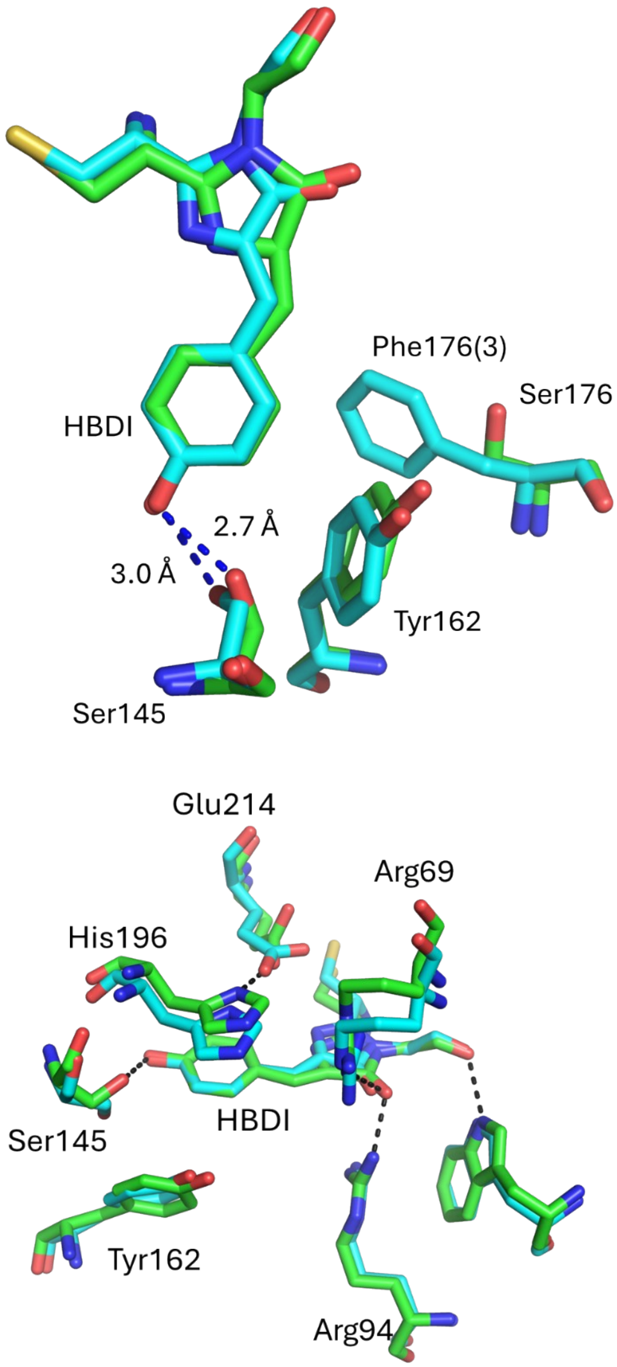

Fig S14 Top. The structure of Kohinoor compared with Padron0.9 (Figure 2a) focused on the region of the phenolic group. The modest changes in structure suggest small changes in the S145 to phenolic OH distances. **Bottom. To highlight changes in the chromophore environment, the comparison in Figure 2a (chain aligned structures) is made with the two chromophores here aligned by overlapping their phenol rings, highlighting structure changes in and around the chromophore.**

Additional References:

(1) Kondo, M.; Heisler, I. A.; Conyard, J.; Rivett, J. P. H.; Meech, S. R. Reactive Dynamics in Confined Liquids: Interfacial Charge Effects on Ultrafast Torsional Dynamics in Water Nanodroplets. *J. Phys. Chem. B* **2009**, *113* (6), 1632–1639. https://doi.org/10.1021/jp808991g.

(2) Roy, P.; Bressan, G.; Gretton, J.; Cammidge, A. N.; Meech, S. R. Ultrafast Excimer Formation and Solvent Controlled Symmetry Breaking Charge Separation in the Excitonically Coupled Subphthalocyanine Dimer. *Angew. Chemie - Int. Ed.* **2021**, *60* (19), 10568–10572. https://doi.org/10.1002/anie.202101572.

(3) Towrie, M.; Grills, D. C.; Dyer, J.; Weinstein, J. A.; Matousek, P.; Barton, R.; Bailey, P. D.; Subramaniam, N.; Kwok, W. M.; Ma, C.; Phillips, D.; Parker, A. W.; George, M. W. Development of a Broadband Picosecond Infrared Spectrometer and Its Incorporation into an Existing Ultrafast Time-Resolved Resonance Raman, UV/Visible, and Fluorescence Spectroscopic Apparatus. *Life Sci. Leg. Georg. Porter* **2006**, *57* (4), 454–467. https://doi.org/10.1142/9781860948930_0009.

(4) Greetham, G. M.; Sole, D.; Clark, I. P.; Parker, A. W.; Pollard, M. R.; Towrie, M. Time-Resolved Multiple Probe Spectroscopy. *Rev. Sci. Instrum.* **2012**, *83* (10). https://doi.org/10.1063/1.4758999.

(5) Tiwari, D. K.; Arai, Y.; Yamanaka, M.; Matsuda, T.; Agetsuma, M.; Nakano, M.; Fujita, K.; Nagai, T. A Fast- and Positively Photoswitchable Fluorescent Protein for Ultralow-Laser-Power RESOLFT Nanoscopy. *Nat. Methods* **2015**, *12* (6), 515–518. https://doi.org/10.1038/nmeth.3362.

(6) Walter, A.; Andresen, M.; Jakobs, S.; Schroeder, J.; Schwarzer, D. Primary Light-Induced Reaction Steps of Reversibly Photoswitchable Fluorescent Protein Padron0.9 Investigated by Femtosecond Spectroscopy. *J. Phys. Chem. B* **2015**, *119* (16), 5136–5144. https://doi.org/10.1021/jp512610q.
